# Sparse data embedding and prediction by tropical matrix factorization

**DOI:** 10.1186/s12859-021-04023-9

**Published:** 2021-02-25

**Authors:** Amra Omanović, Hilal Kazan, Polona Oblak, Tomaž Curk

**Affiliations:** 1 Faculty of Computer and Information Science, University of Ljubljana, Večna pot 113, 1000 Ljubljana, Slovenia; 2 Department of Computer Engineering, Antalya Bilim University, Çıplaklı, Akdeniz Blv. No:290/A, 07190 Antalya, Turkey

**Keywords:** Data embedding, Matrix factorization, Tropical factorization, Sparse data, Matrix completion, Tropical semiring

## Abstract

**Background:**

Matrix factorization methods are linear models, with limited capability to model complex relations. In our work, we use tropical semiring to introduce non-linearity into matrix factorization models. We propose a method called *Sparse Tropical Matrix Factorization* (STMF) for the estimation of missing (unknown) values in sparse data.

**Results:**

We evaluate the efficiency of the STMF method on both synthetic data and biological data in the form of gene expression measurements downloaded from The Cancer Genome Atlas (TCGA) database. Tests on unique synthetic data showed that STMF approximation achieves a higher correlation than non-negative matrix factorization (NMF), which is unable to recover patterns effectively. On real data, STMF outperforms NMF on six out of nine gene expression datasets. While NMF assumes normal distribution and tends toward the mean value, STMF can better fit to extreme values and distributions.

**Conclusion:**

STMF is the first work that uses tropical semiring on sparse data. We show that in certain cases semirings are useful because they consider the structure, which is different and simpler to understand than it is with standard linear algebra.

## Background

Matrix factorization methods are getting increasingly popular in many research areas [1–3]. These methods generate linear models, which cannot model complex relationships. Our work focuses on incorporating non-linearity into matrix factorization models by using tropical semiring.

The motivation for using tropical matrix factorization can be seen in the classic example of movie rating data, where a users-by-movies matrix contains the rating users assigned to movies. In standard matrix factorization methods, it is assumed that a user’s final rating is a linear combination of some factors (a person likes some movie because of the director, the genre, the lead actor, etc.). But it is also possible that some factor is so dominant that all others are irrelevant. An example given for the Latitude algorithm [4], a person likes all Star Wars movies irrespective of actors or directors, shows that using the $\max$ operator instead of the sum might produce a better model.

We develop a method for the prediction of missing (unknown) values, called *Sparse Tropical Matrix Factorization* (STMF). We evaluate its performance on the prediction of gene expression measurements from The Cancer Genome Atlas Research Network (TCGA) database. We show that the newly defined operations can discover patterns, which cannot be found with standard linear algebra.

## Related work

Matrix factorization is a data embedding model which gives us a more compact representation of the data and simultaneously finds a latent structure. The most popular example is the non-negative matrix factorization (NMF) [5], where the factorization is restricted to the matrices with non-negative entries. This non-negativity in resulting factor matrices makes the results easier to interpret. One of the applications of matrix factorization methods is for recommender systems, where users and items are represented in a lower-dimensional latent space [6]. Binary matrix factorization (BMF) [7, 8] is a variant rooted from NMF where factor matrices are binary, while probabilistic non-negative matrix factorization (PMF) [9] models the data as a multinomial distribution. MMDNMF [10] is a supervised NMF method, which minimizes the maximum distance within-class and maximizes the minimum distance between-class. Integrative approaches, which use standard linear algebra to simultaneously factorize multiple data sources and improve predictive accuracy, are reviewed in [11]. Multi-omic and multi-view clustering methods like MultiNMF [12], Joint NMF [13], PVC [14], DFMF [15], MDNMF [16] and iONMF [17] can be used for data fusion of multiple data sources.

Lately, subtropical semiring $(\max , \cdot )$ gained interest in the field of machine learning, since it can discover interesting patterns [18, 19]. By taking the logarithm of the subtropical semiring, we obtain the tropical semiring $(\max , +)$ [20]. Although these two semirings are isomorphic, the factorization in tropical semiring works differently than the factorization in subtropical semiring. The Cancer algorithm [20] works with continuous data, performing subtropical matrix factorization (SMF) on the input matrix. Two main components of the algorithm are: iteratively updating the rank-1 factors one-by-one and approximate the max-times reconstruction error with a low-degree polynomial. Latitude algorithm [4] combines NMF and SMF, where factors are interpreted as NMF features, SMF features or as mixtures of both. This approach gives good results in cases where the underlying data generation process is a mixture of the two processes. In [21] authors used subtropical semiring as part of a recommender system. We can consider their method to be a particular kind of neural network. Le Van et al. [22] presented a single generic framework that is based on the concept of semiring matrix factorization. They applied the framework on two tasks: sparse rank matrix factorization and rank matrix tiling.

De Schutter & De Moor [23] presented a heuristic algorithm to compute factorization of a matrix in the tropical semiring, which we denote as *Tropical Matrix Factorization* (TMF). They use it to determine the minimal system order of a discrete event system (DES). In the last decades, there has been an increase of interest in this research area, and DES is modeled as a max-plus-linear (MPL) system [24, 25]. In contrast to TMF where approximation error is reduced gradually, convergence is not guaranteed in the Cancer algorithm. Both Cancer and TMF return factors that encode the most dominant components in the data. However, by their construction, they cannot be used for prediction tasks in different problem domains, such as predicting gene expression. In contrast with the NMF method and its variants, which require non-negative data, TMF can work with negative values.

Hook [26] reviewed algorithms and applications of linear regression over the max-plus semiring, while Gärtner and Jaggi [27] constructed a tropical analogue of support vector machines (SVM), which can be used to classify data into more than just two classes compared to the classical SVM. Zhang et al. [28] in their work establish a connection between neural networks and tropical geometry. They showed that linear regions of feedforward neural networks with rectified linear unit activation correspond to vertices of polytopes associated with tropical rational functions. Therefore, to understand specific neural networks, we need to understand relevant tropical geometry. Since one goal in biology is not just to model the data, but also to understand the underlying mechanisms, the matrix factorization methods can give us a more straightforward interpretation than neural networks. The GCN-MF framework [29] uses matrix factorization to combine embeddings and *Graph Convolutional Network* (GCN) using standard linear algebra. The authors state that matrix factorization only utilizes the linear relationship between entities. When data is more complex, the matrix factorization method cannot identify non-linear relationships. Since deep learning uses non-linear functions and layer combinations, neural networks can learn more complex data patterns. In our work, instead of introducing deep learning, we address the issue of non-linearity with tropical semiring.

In our work, we answer the question stated in Cancer: can tropical factorization be used, in addition to data analysis, also in other data mining and machine learning tasks, e.g. matrix completion? We propose a method STMF, which is based on TMF, and it can simultaneously predict missing values, i.e. perform matrix completion. In Table 1 we compare the most relevant methods for our work. To the best of our knowledge STMF is the only method which performs prediction tasks in tropical semiring. STMF introduces non-linearity into matrix factorization models, which enables discovering the most dominant patterns, leading to a more straightforward visual interpretation compared to other methods for missing value prediction.

**Table 1:** A comparison between different matrix factorization methods

	Arithmetic	Data sources	Prediction tasks	Convergence
NMF [5], BMF [7], PMF [9], MMDNMF [10]	Standard	Single	Yes	Yes
DFMF [15], iONMF [17], MDNMF [16]	Standard	Multiple	Yes	Yes
Latitude [4]	Standard & Subtropical	Single	No	No
Cancer [20]	Subtropical	Single	No	No
TMF [23]	Tropical	Single	No	Yes
STMF	Tropical	Single	Yes	Yes

## Methods

### Tropical semiring and factorization

Now, we give some formal definitions regarding the tropical semiring. The $(\max ,+)$ *semiring* or *tropical semiring* ${\mathbb {R}}_{\max }$, is the set ${\mathbb {R}} \cup \{-\infty \}$, equipped with $\max$ as addition ($\oplus$), and $+$ as multiplication ($\otimes$). For example, $2\oplus 3=3$ and $1\otimes 1=2$. On the other hand, in the *subtropical semiring* or $(\max ,\times )$ *semiring*, defined on the same set ${\mathbb {R}} \cup \{-\infty \}$, addition ($\max$) is defined as in the tropical semiring, but the multiplication is the standard multiplication ($\times$). Throughout the paper, symbols $+$ and − refer to standard operations of addition and subtraction. Tropical semiring can be used for optimal control [30], asymptotics [31], discrete event systems [32] or solving a decision problem [33]. Another example is the well-known game Tetris, which can be linearized using the $(\max ,+)$ semiring [34].

Let ${\mathbb {R}}_{\max }^{m \times n}$ define the set of all $m \times n$ matrices over tropical semiring. For $A\in {\mathbb {R}}_{\max }^{m \times n}$ we denote by $A_{ij}$ the entry in the *i*-th row and the *j*-th column of matrix *A*. We denote the *sum of matrices* $A, B \in {\mathbb {R}}_{\max }^{m \times n}$ as $A\oplus B \in {\mathbb {R}}_{\max }^{m \times n}$ and define its entries as

$$\begin{aligned} (A \oplus B)_{ij} = A_{ij} \oplus B_{ij} = \max \{A_{ij}, B_{ij}\}, \end{aligned}$$

$i=1,\ldots ,m$, $j=1,\ldots ,n$. The *product of* matrices $A \in {\mathbb {R}}_{\max }^{m \times p}$, $B \in {\mathbb {R}}_{\max }^{p \times n}$ is denoted by $A\otimes B \in {\mathbb {R}}_{\max }^{m \times n}$ and its entries are defined as

$$\begin{aligned} (A \otimes B)_{ij} = \bigoplus \limits _{k=1}^{p} A_{ik} \otimes B_{kj} = \max \limits _{1\le k \le p}\{A_{ik} + B_{kj}\}, \end{aligned}$$

$$i=1,\ldots ,m$$, $$j=1,\ldots ,n$$.

*Matrix factorization over a tropical semiring* is a decomposition of a form $R= U \otimes V$, where $R \in {\mathbb {R}}_{\max }^{m \times n}$, $U \in {\mathbb {R}}_{\max }^{m \times r}$, $V \in {\mathbb {R}}_{\max }^{r \times n}$ and $r \in {\mathbb {N}}_0$. Since for small values of *r* such decomposition may not exist, we state tropical matrix factorization problem as: given a matrix *R* and factorization rank *r*, find matrices *U* and *V* such that

1 $$\begin{aligned} R\cong U \otimes V. \end{aligned}$$

To implement a tropical matrix factorization algorithm, we need to know how to solve tropical linear systems. Methods for solving linear systems over tropical semiring differ substantially from methods that use standard linear algebra [34].

We define the *ordering* in tropical semiring as $z \preceq w$ if and only if $z \oplus w=w$ for $z,w \in {\mathbb {R}}_{\max }$, and it induces the ordering on vectors and matrices over tropical semiring entry-wise. For $A\in {\mathbb {R}}_{\max }^{m \times n}$ and $c=[c_k] \in {\mathbb {R}}_{\max }^{m}$ the system of linear inequalities $A\otimes x \preceq c$ always has solutions and we call the solutions of $A \otimes x \preceq c$ the *subsolutions* of the linear system $A\otimes x=c$. The greatest subsolution $x=[x_1\, x_2\, \ldots x_n]^T$ of $Ax =c$ can be computed by

2 $$\begin{aligned} x_i = \min _{1 \le j \le m} (c_j - A_{ji}) \end{aligned}$$

for $i=1,2,\ldots ,n$. We will use (2) in a column-wise form to solve the matrix equations.

TMF starts with an initial guess for the matrix *U* in (1), denoted by $U_0$ and then computes *V* as the greatest subsolution of $U_0 \otimes X = R$. Then authors use the iterative procedure by selecting and adapting an entry of *U* or *V* and recomputing it as the greatest subsolution of $Y \otimes V = R$ and $U \otimes X = R$, respectively. The *b*-*norm* of matrix *W*, defined as the objective function $||W||_b=\sum _{i,j}\vert W_{ij} \vert$ is used to minimize the approximation error $\Vert R - U \otimes V\Vert _b$.

### Our contribution

In our work, we implement and modify TMF so that it can be applied in data mining tasks. We propose a sparse version of TMF, which can work with missing values.

In *Sparse Tropical Matrix Factorization* (STMF), which is available on https://github.com/Ejmric/STMF, we update the factor matrices *U* and *V* based on the selected given entry of the input data matrix *R* to predict the missing values in *R*. In Algorithm 1, we present the pseudocode of STMF in which for each given entry (*i*, *j*) of *R* we first update *U* and *V* based on the element from the *i*th row of the left factor *U* (ULF, see Algorithm 2). If the update of the factors does not improve the approximation of *R*, then we update *U* and *V* based on the element from the *j*th column of the right factor *V* (URF, see Algorithm 3). 
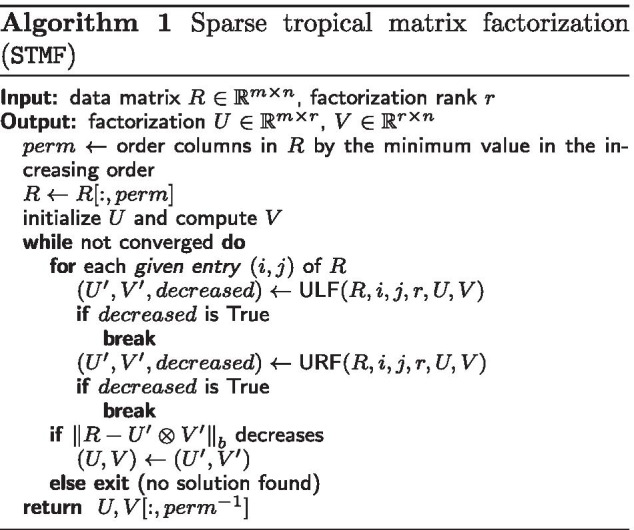


Algorithms ULF and URF differ from the corresponding TMF’s versions in the way they solve linear systems. Since some of the entries of matrix *A* are not given, we define $(\min , +)$ matrix multiplication $\otimes ^{*}$ as

$$\begin{aligned} (A \otimes ^{*} B)_{ij} = \min \limits _{A_{ik}, B_{kj} \text {are given}}\{A_{ik} + B_{kj}\} \end{aligned}$$

for matrices $A \in {\mathbb {R}}_{\max }^{m \times p}$ and $B \in {\mathbb {R}}_{\max }^{p \times n}$, $i=1,\ldots ,m$, $j=1,\ldots ,n$. Newly-defined operator $\otimes ^*$ can be seen as a generalization of Eq. (2), and it is used for solving linear systems by skipping unknown values. We assume that at least one element in each row/column is known. 
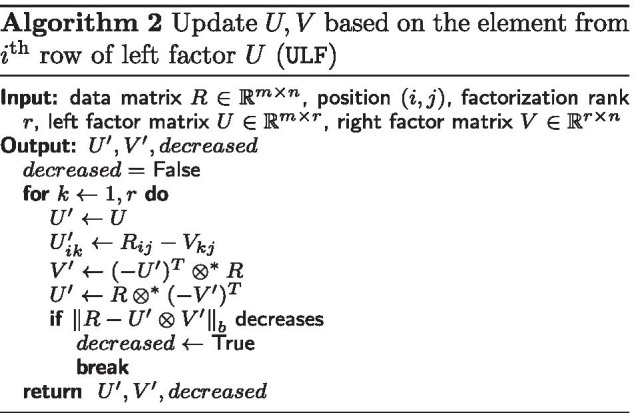

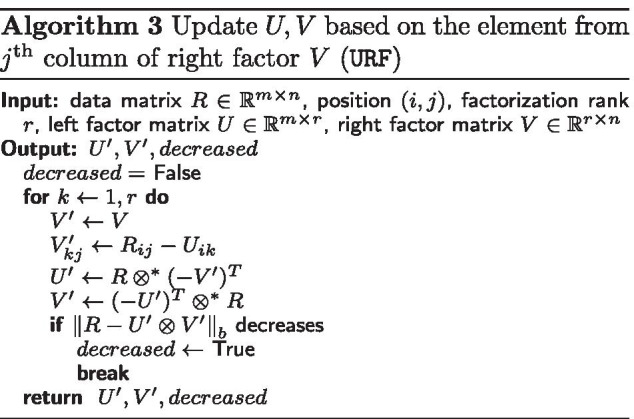


Among the different matrix initialization strategies, we obtained the best performance with Random Acol strategy [15, 35]. Random Acol computes each column of the initialized matrix *U* as an element-wise average of a random subset of columns of the data matrix *R*. It is a widely used method for initializations in matrix factorization methods since it gives better insight into the original data matrix than simple random initialization.

In contrast to Cancer, where convergence is not guaranteed, the update rules of STMF, similar to TMF, gradually reduce the approximation error. This is ensured by the fact that factor matrices *U* and *V* are only updated in the case when $\left\Vert R - U \otimes V \right\Vert _b$ monotonously decreases.

### Distance correlation

It is well known that Pearson and Spearman correlation coefficients can misrepresent non-linear relationships [36]. Since in real data, we often deal with non-linearity, our choice is to use so-called *distance correlation*. Distance correlation [37] is a straightforward measure of association that uses the distances between observations as part of its calculation. It is a better alternative for detecting a wide range of relationships between variables.

Let *X* and *Y* be the matrices each with *n* rows and *A* and *B* their matrices of Euclidean distances with the row/column means subtracted, and grand mean added. After matrix centering the *distance covariance* $V_{xy}$ is defined as

$$\begin{aligned} V_{XY}^2 = \frac{1}{n^2}\sum _{i,j=1}^{n} A_{ij} B_{ij}, \end{aligned}$$

and distance correlation $\mathrm{dcor}$ as

$$\begin{aligned} {\mathrm{dcor}}(X,Y) = \sqrt{\frac{V_{XY}^2}{V_X V_Y}}, \end{aligned}$$

where $V_X$ and $V_Y$ represent distance variances of matrices *X* and *Y*. Distance correlation is 0 only if the two corresponding variables are independent.

Distance correlation cannot be used to compare specific rows between *X* and *Y*, because it requires the entire matrix to be centered first. In such cases we use Euclidean norm between rows of centered original and rows of centered approximated data.

### Synthetic data

We create two types of synthetic datasets of rank 3: one smaller of size $200 \times 100$ and five larger of size $500 \times 300$. We use the $(\max , +)$ multiplication of two random non-negative matrices sampled from a uniform distribution over [0, 1) to generate each synthetic dataset.

### Real data

We download the preprocessed TCGA data [11] for nine cancer types, where for each cancer type three types of omic data are present: gene expression, methylation and miRNA data. We transpose the data sources, so that in each data source, the rows represent patients and columns represent features. The first step of data preprocessing is to take the subset of patients for which we have all three data sources. In our experiments we use only gene expression data. After filtering the patients, we substitute each gene expression value *x* in the original data with the $\log _2(x+1)$. With log-transformation, we make the gene expression data conform more closely to the normal distribution, and by adding one, we reduce the bias of zeros. We also perform polo clustering, which is an optimal linear leaf ordering [38], to re-order rows and columns on the preprocessed data matrix. Polo clustering results in a more interpretative visualization of factor matrices.

Next, we use feature agglomeration to merge similar genes by performing clustering [39]. We use Ward linkage and split genes into 100 clusters (see Additional file 1: Figure S 24), the center of each cluster representing a *meta-gene*. With this approach, we minimize the influence of non-informative, low variance genes on distance calculations and reduce the computational requirements.

**Table 2:** Size of gene expression data in the form of $patients \times meta\text {-}genes$ for eight cancer subtypes, and for the subset of PAM50 genes in BIC

Cancer subtype	Size
Acute Myeloid Leukemia (AML)	$171 \times 100$
Colon Adenocarcinoma (COLON)	$221 \times 100$
Glioblastoma Multiforme (GBM)	$274 \times 100$
Liver Hepatocellular Carcinoma (LIHC)	$410 \times 100$
Lung Squamous Cell Carcinoma (LUSC)	$344 \times 100$
Ovarian serous cystadenocarcinoma (OV)	$291 \times 100$
Skim Cutaneous Melanoma (SKCM)	$450 \times 100$
Sarcoma (SARC)	$261 \times 100$
Breast Invasive Carcinoma (BIC)	$541 \times 50$

For Breast Invasive Carcinoma (BIC), we do not perform feature agglomeration since a list of 50 genes, called PAM50 [40], classify breast cancers into one of five subtypes: LumA, LumB, Basal, Her2, and Normal [41, 42], resulting in our BIC data matrix of size $541 \times 50$. These five subtypes differ significantly in the expression of only a few genes in BIC data, which leads to the value close to zero for silhouette score [43] (see Additional file 1: Figure S 22). The sizes of the final nine datasets are listed in Table 2.

### Performance evaluation

Since STMF is the first work in tropical semiring, which performs matrix completion, we choose NMF as a baseline method because it represents the original matrix factorization method using standard linear algebra. In contrast, other methods from Table 1, which use standard linear algebra, are extensions of NMF. Additionally, we provide running time (Table 3) and distance correlation (Table 4) results for PMF because it represents the advanced version of NMF that is suitable for performing prediction task on a single dataset.

Experiments were performed for varying values of the factorization rank. The smaller synthetic dataset experiments were run 10 times, with 500 iterations each, and on larger synthetic datasets, experiments were run 50 times, with 500 iterations each. Experiments for real data were run five times, with 500 iterations. For both datasets, we mask randomly and uniformly $20 \%$ of data as missing, which we then use as a test set to evaluate the tested methods. We assume that in a typical dataset, data will be missing uniformly at random. The remaining $80 \%$ represent the training set. We choose a rank based on the approximation error on training data, which represents a fair/optimal choice for both methods, STMF and NMF so that we can compare them, knowing both of them to have the same number of parameters.

We compute the distance correlation and Euclidean norm between the original and approximated data matrix to evaluate the predictive performance.

## Results

First, we use synthetic data to show the correctness of the STMF algorithm. We use the smaller dataset to show that STMF can discover the tropical structure. The larger datasets are needed to show how the order of rows and columns affects the result. We then apply it to real data to compare the performance and interpretability of models obtained with STMF and NMF.

### Synthetic data

**Fig. 1:**
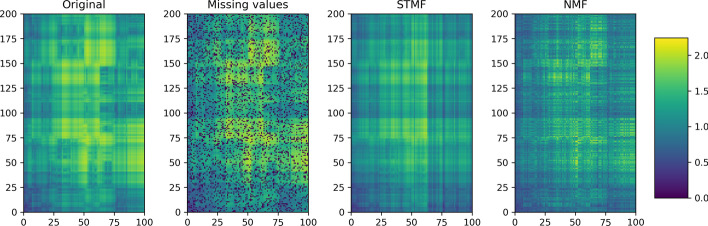
A comparison between STMF’s and NMF’s predictions of best rank 4 approximations on $200 \times 100$ synthetic $(\max ,+)$ matrix with $20\%$ missing values

**Fig. 2:**
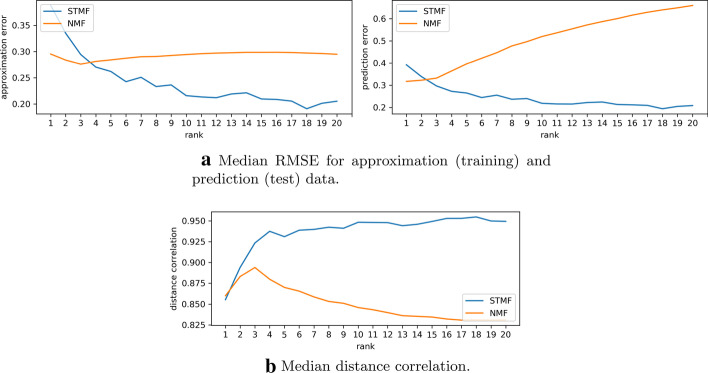
Comparison of STMF (blue) and NMF (orange) on synthetic $(\max ,+)$ matrix of size $200 \times 100$ and rank 3

The objective of synthetic experiments is to show that STMF can identify the $(\max ,+)$ structure when it exists. Even on a relatively small $200 \times 100$ matrix results show that NMF cannot successfully recover extreme values compared to STMF, see Fig. 1. NMF and PMF tend towards zero values, which results in a blurry visualization of the approximation matrices (Additional file 1, Subsection 1.1.1). The values of the matrices predicted by NMF and PMF that arise from missing values are much smaller that the values on the same positions in the matrix predicted by STMF. This implies that STMF is more efficient when predicting extreme values. This effect is even more pronounced when the missing values are not missing at random (Additional file 1, Subsection 1.1.2), supporting previous reports by Lin and Boutros [44]. STMF demonstrates to be more robust to the choice of sampling strategy of missing values. As the results show STMF achieves a smaller prediction root-mean-square-error (RMSE) and higher distance correlation (Fig. 2).

Experiments on synthetic data show that changing the execution order of URF and ULF in the computation of STMF does not affect the result of the algorithm.

**Fig. 3:**
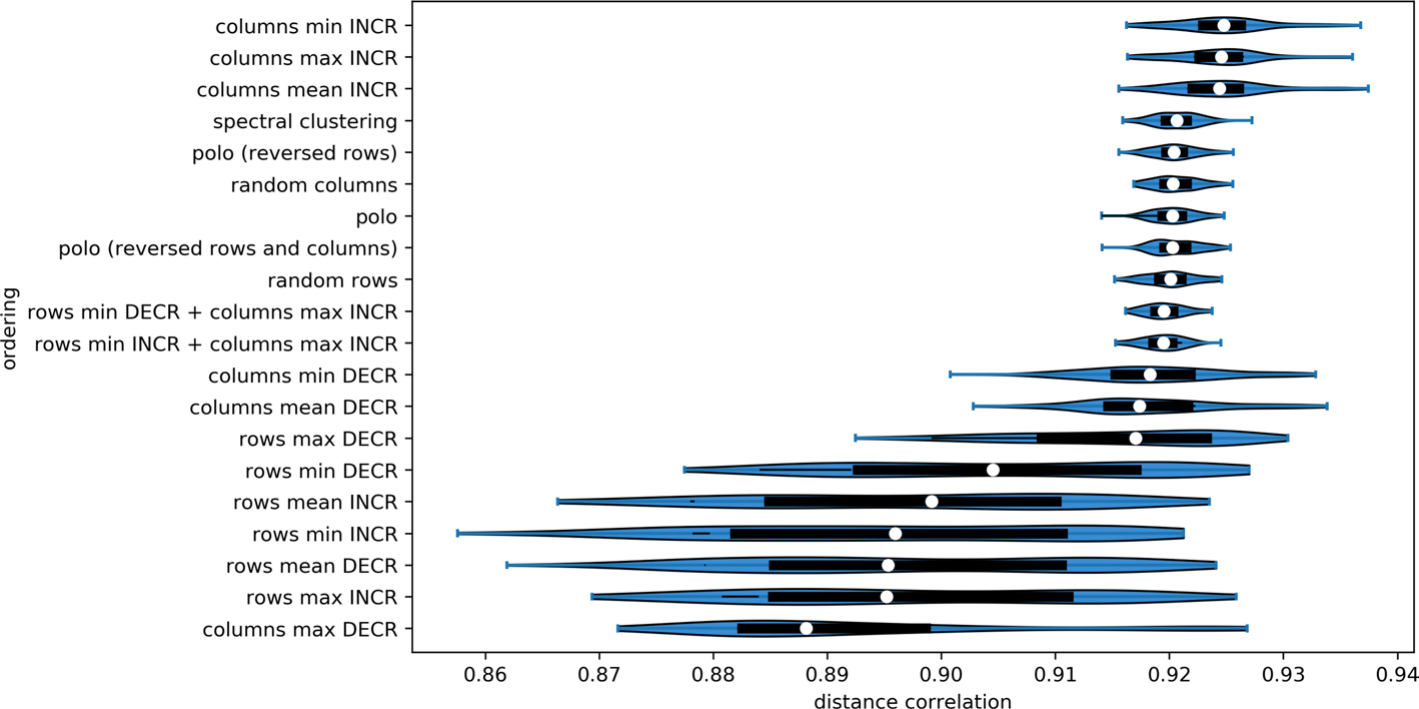
Effect of ordering strategy on achieved distance correlation by STMF, on $500 \times 300$ synthetic $(\max , +)$ matrix. Top three performing strategies order columns by increasing values

The result of STMF depends on the order of matrix entries. We perform different types of permutation techniques to order columns and rows on five large synthetic datasets (see Additional file 1: Figure S 19). Top three strategies are to sort columns by increasing values of their minimum, maximum, and mean value (Fig. 3). Moreover, in four out of five datasets, the best results were obtained by ordering columns in increasing order by their minimum value (see Additional file 1: Figure S 20). This strategy represents the first step of STMF method (Algorithm 1).

### Real data

**Fig. 4:**
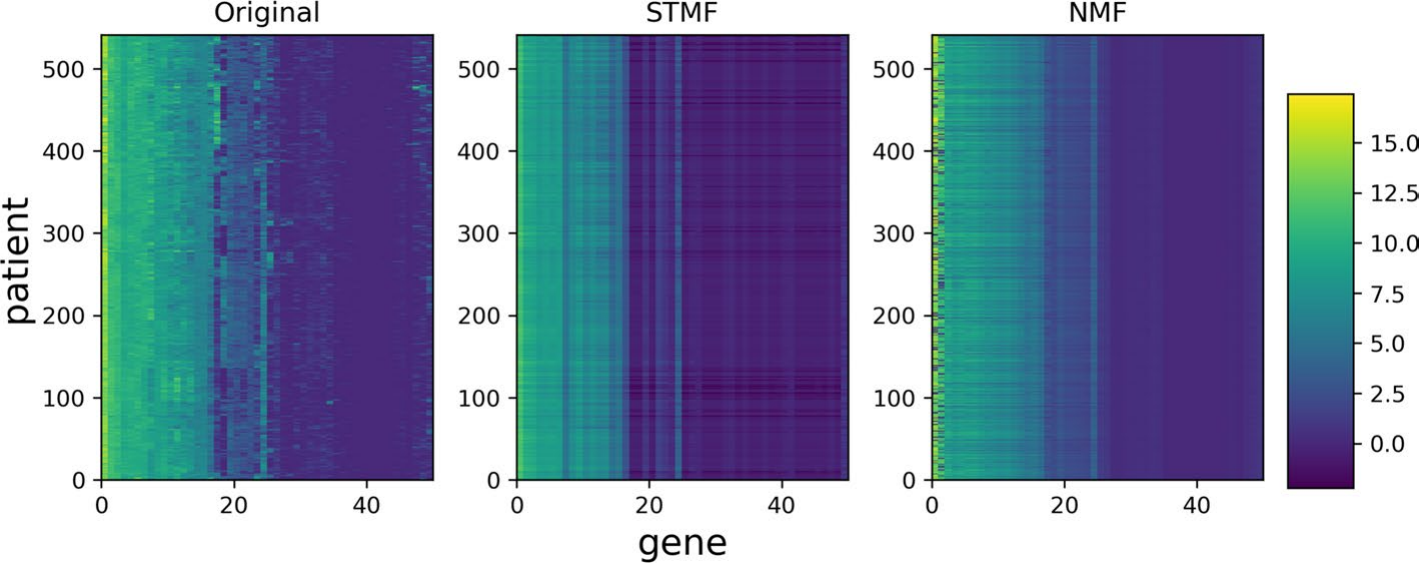
Best rank 3 approximation matrices $R_{\texttt {STMF}}$ and $R_{\texttt {NMF}}$ from STMF and NMF on the prediction of the gene expression signal on Breast Invasive Carcinoma (BIC) tumor

Figure 4 shows the results on BIC matrix, with PAM50 genes and 541 patients. Our findings confirm that STMF expresses some extreme values. We see that STMF successfully recovers large values, while NMF has the largest error where gene expression values are high. Note that NMF tends towards the mean value. Half of the original data is close to zero (plotted in dark blue), which is a reason that NMF cannot successfully predict high (yellow) values. For all other datasets approximation matrices are available in Additional file 1, Section 2.

**Fig. 5:**
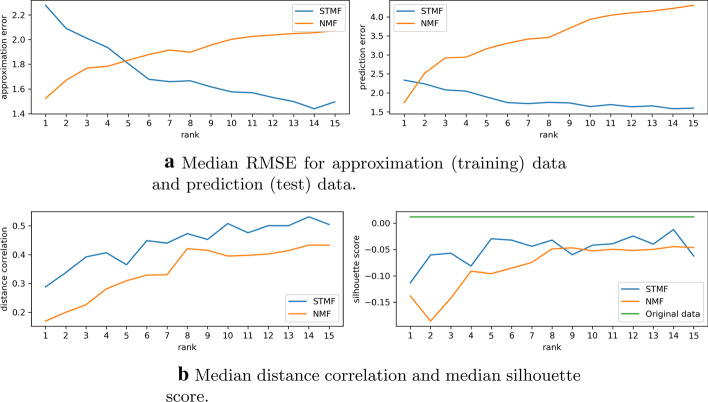
Comparison of performance of STMF (blue) and NMF (orange) on BIC matrix

In Fig. 5a we see that NMF has smaller approximation error than STMF, but larger prediction error. So, NMF better approximates/fits the data, but STMF is not prone to overfitting, since its prediction error is smaller. On the other hand, in Fig. 5b, STMF has better distance correlation and silhouette score values; silhouette score for PMF is shown in Additional file 1: Figure S 23. Thus, STMF can find clusters of patients with the same subtype better than NMF, which tends to describe every patient by the mean values in data. For all other datasets similar graphs are available in Additional file 1, Section 2.

**Fig. 6:**
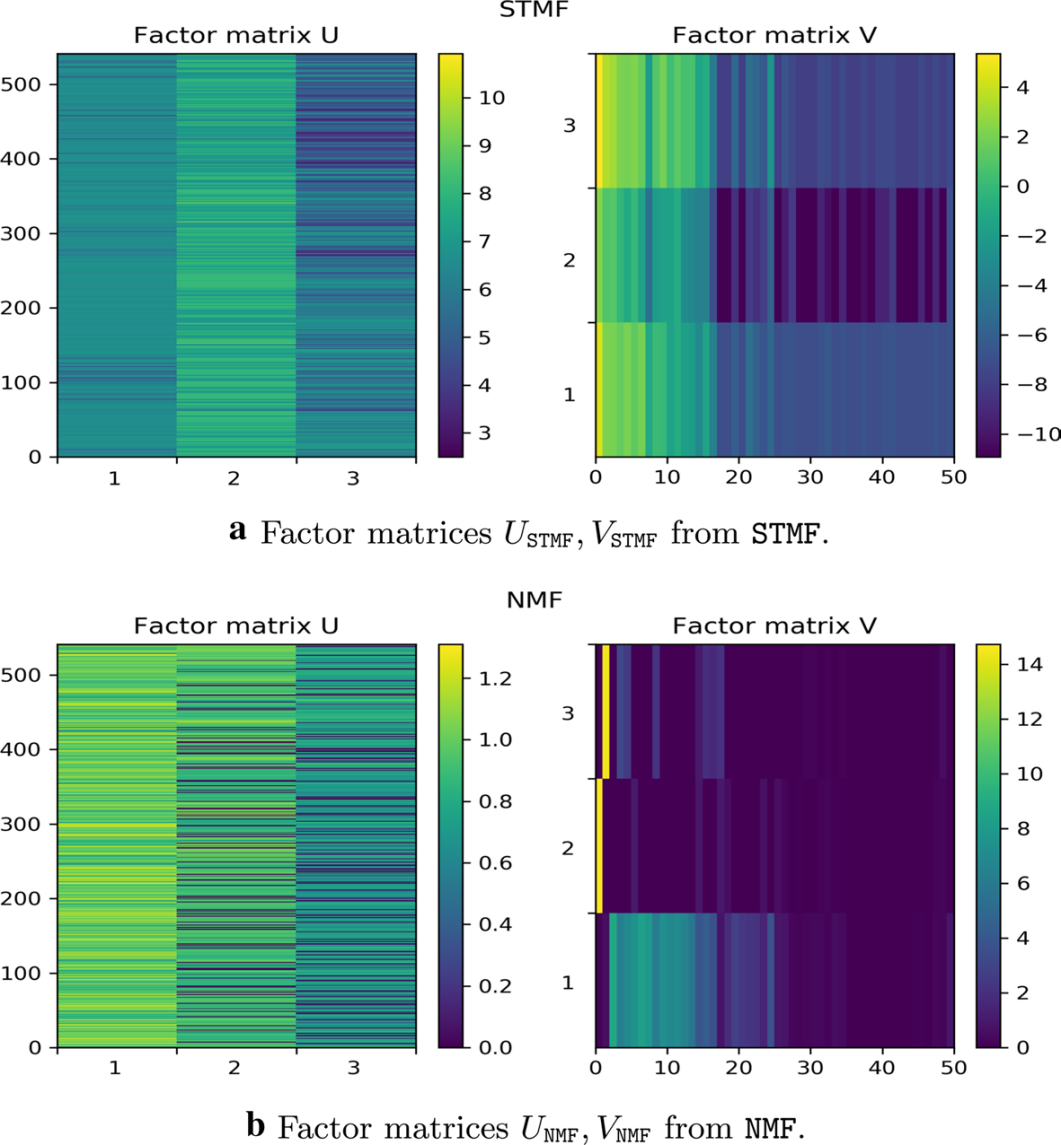
Factor matrices $U_{\texttt {STMF}}, V_{\texttt {STMF}}$ and $U_{\texttt {NMF}}, V_{\texttt {NMF}}$ obtained by STMF and NMF algorithms, respectively, on BIC matrix

The rank three factor matrices of the BIC matrix (see Fig. 4) are illustrated in Fig. 6, where we denote STMF’s factor matrices by $U_{\texttt {STMF}}, V_{\texttt {STMF}}$, and NMF’s factor matrices by $U_{\texttt {NMF}}, V_{\texttt {NMF}}$. We see that these factor matrices are substantially different. Basis factor $V_{\texttt {STMF}}$ (first and third row) is visually the most similar to the original matrix than any other factor alone. Factor $V_{\texttt {STMF}}$ detects low and high values of gene expression, while factor $V_{\texttt {NMF}}$ detects high values in the first two columns (second and third row, respectively) and low values in remaining columns (first row). Coefficient factors $U_{\texttt {STMF}}$ and $U_{\texttt {NMF}}$ contribute to a good approximation of the original matrix. For all other datasets factor matrices are available in Additional file 1, Section 2.

**Fig. 7:**
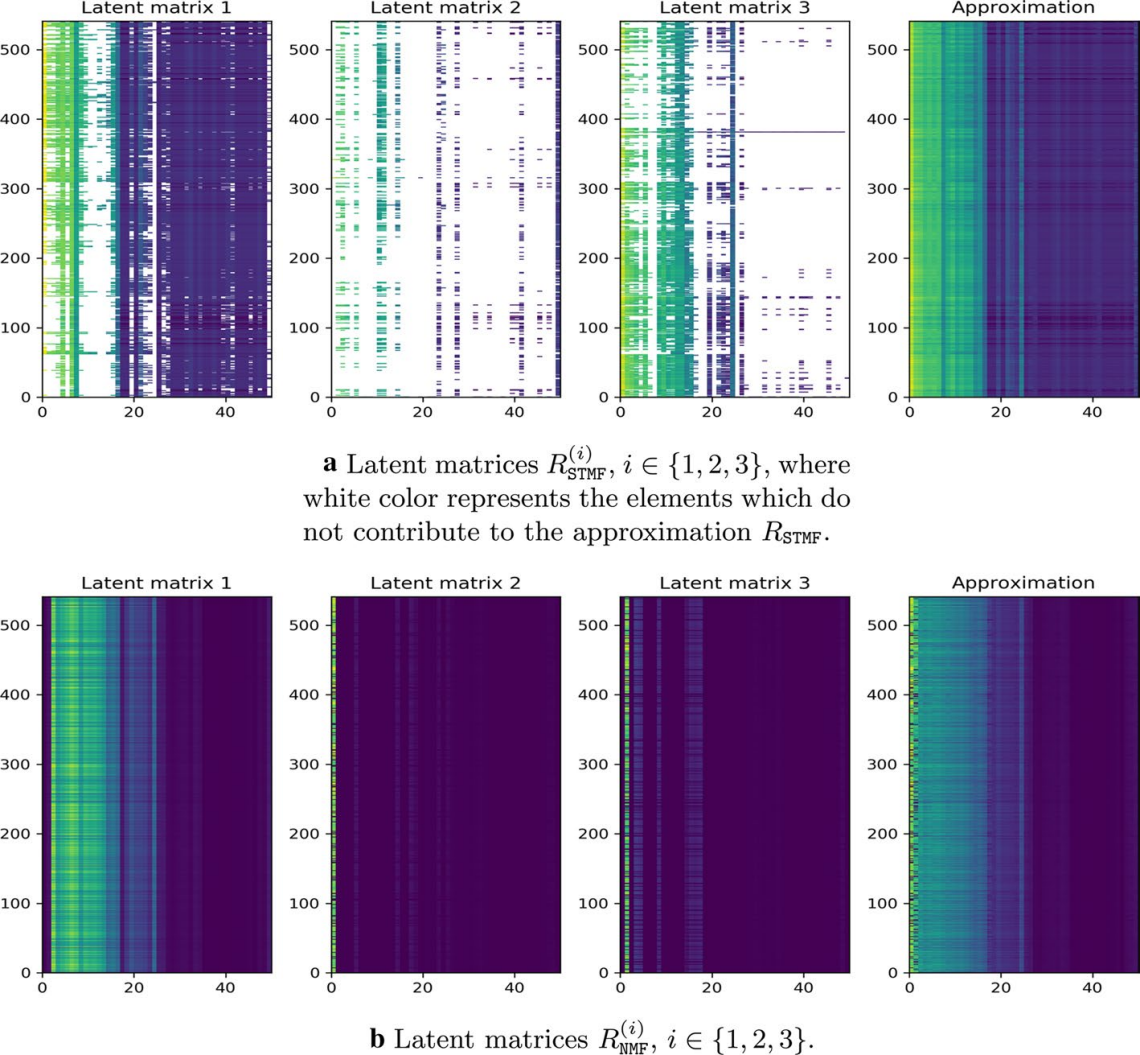
STMF’s and NMF’s latent matrices on BIC matrix

To see which part of data is explained by which factorization rank, we define a latent matrix $R^{(i)}$ as a reconstruction using only one latent component from the approximation matrix, where $i \in \{1,\ldots , r\}$, and *r* is the factorization rank. $R^{(i)}$ can be seen as a projection on the direction of the *i*-th factor. For example, $R_{\texttt {STMF}}^{(1)}$ matrix in Fig. 7a is a result of the (max, +) product, which represent sums of each pair of elements, of the first column of $U_{\texttt {STMF}}$ and the first row of $V_{\texttt {STMF}}$ (Fig. 6). In the case of NMF, instead of sum, there is multiplication (see Fig. 7b). If we compute an element-wise maximum of all $R_{\texttt {STMF}}^{(i)}$ we get the $R_{\texttt {STMF}}$, while element-wise sum of all $R_{\texttt {NMF}}^{(i)}$ results in $R_{\texttt {NMF}}$. In this way, we see which latent matrix $R^{(i)}$ explains which part of the data. On the BIC matrix, we see that both methods, STMF, and NMF, describe most of the data with the first latent matrix (Fig. 7). For all other datasets latent matrices are available in Additional file 1, Section 2.

In Table 4 we present the results of experiments on nine datasets listed in Table 2. We see that STMF outperforms NMF on six out of nine datasets, while NMF achieves better results on the LUSC, SKCM and SARC datasets. When we add to the comparison the PMF method, which is the probabilistic version of NMF, it outperforms STMF and NMF on five datasets, but there is no statistically significant difference between the three methods according to the critical difference (CD) method by Demšar [45] (see Additional file 1: Figure S 21).

Solving linear systems using $\otimes ^{*}$ emphasizes the low (blue) and high (yellow) gene expression values of patients in Fig. 4. In this way, STMF can, in some cases, recover better the original data, while NMF’s results are diluted. However, a limitation of STMF compared to NMF is in its computational efficiency (Table 3).

**Table 3:** Average running times in seconds with the best choice of rank *r* for different matrix factorization methods on nine datasets

Dataset	Rank *r*	STMF [s]	NMF [s]	PMF [s]
AML	3	117.953	0.336	0.028
COLON	3	153.398	0.312	0.864
GBM	3	191.204	0.353	1.996
LIHC	2	236.655	0.467	1.794
LUSC	3	239.329	0.456	3.538
OV	4	251.328	0.336	2.159
SKCM	3	310.401	0.395	6.309
SARC	3	186.475	0.398	0.215
BIC	3	221.669	0.526	0.248

**Table 4:** Distance correlations with the best choice of rank *r* for different matrix factorization methods on nine datasets

Dataset	Rank *r*	STMF			NMF	PMF
		Min.	Median	Max.		
AML	3	0.650	**0**.831*	0.845	0.636	0.623
COLON	3	0.585	**0**.**647**	0.688	0.586	0.707*
GBM	3	0.684	**0**.702*	0.762	0.325	0.330
LIHC	2	0.493	**0**.**515**	0.588	0.311	0.649*
LUSC	3	0.498	0.562	0.731	**0**.**697**	0.799*
OV	4	0.420	**0**.569*	0.601	0.347	0.563
SKCM	3	0.480	0.521	0.605	**0**.**633**	0.808*
SARC	3	0.493	0.584	0.610	**0**.649*	0.588
BIC	3	0.350	**0**.**392**	0.531	0.227	0.427*

Result of best method in the comparison between STMF and NMF shown in bold. Best result among all three methods (STMF, NMF, PMF) indicated by asterisk

**Fig. 8:**
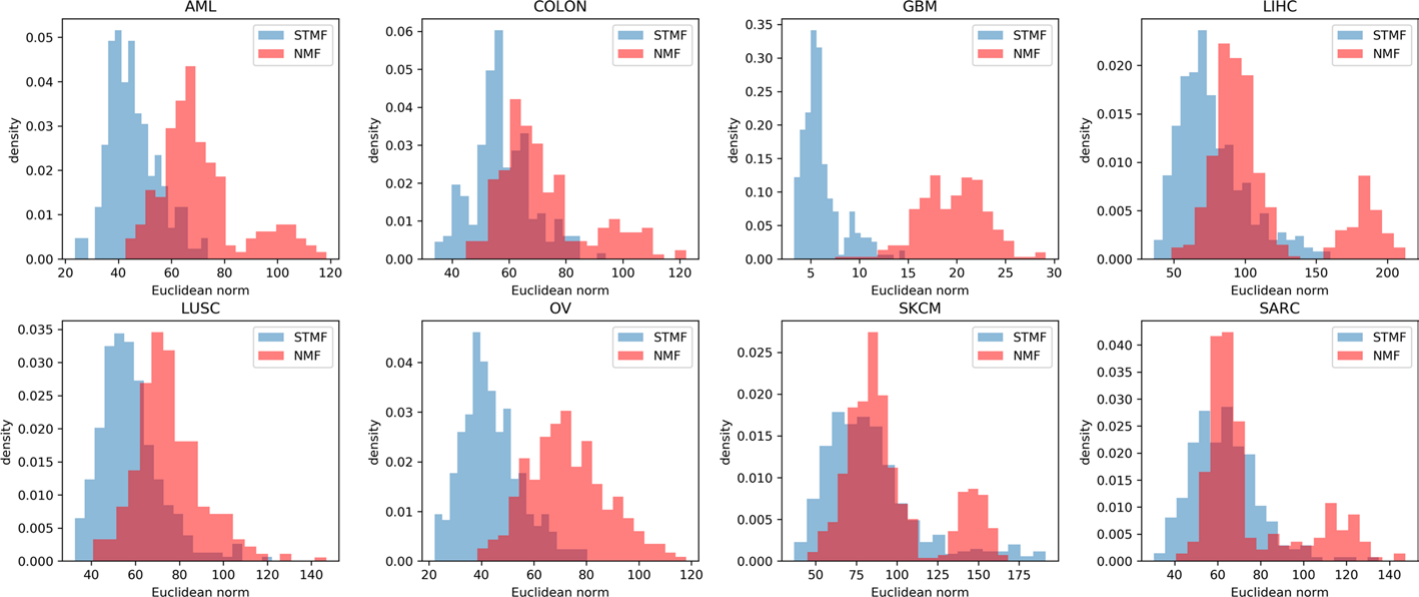
Euclidean norm of difference between centered original data and centered approximations of rank *r* (chosen in Table 4) for different datasets

In Fig. 8 we plot the distribution of Euclidean norm of difference between centered original data and centered approximations of rank *r* (chosen in Table 4) for different datasets. We see that even if we use another metric like Euclidean norm, computed for each row (patient) separately, results still show that STMF outperforms NMF, as it is shown in Table 4 using distance correlation.

**Fig. 9:**
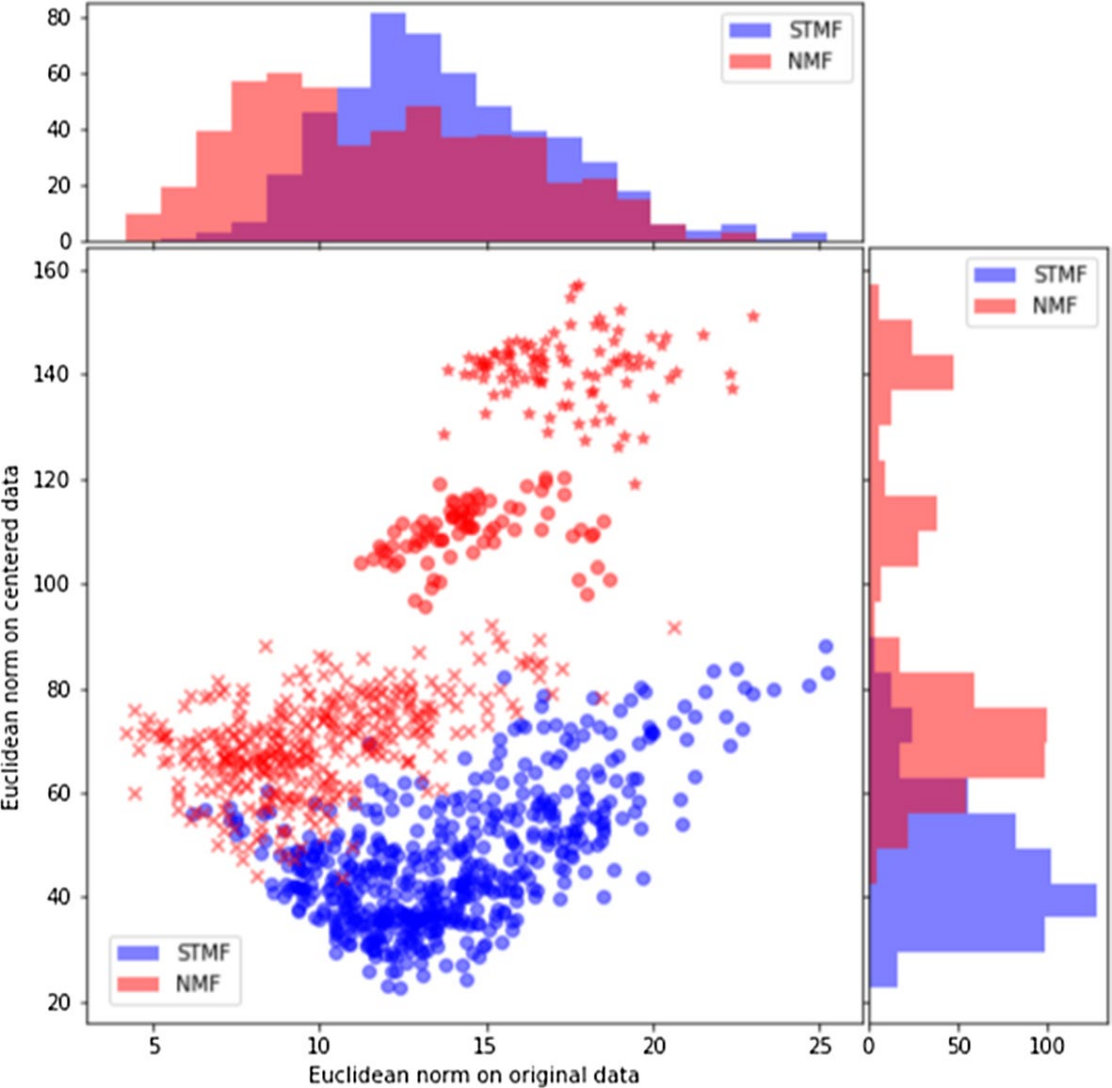
Euclidean norm of difference between original BIC data and approximations (*x*-axis) and Euclidean norm of difference between centered original data and centered approximations (*y*-axis)

In Fig. 9 we explore the difference between the original, approximated and centered BIC dataset. For every row (patient) we present the Euclidean norm of the difference between the rows in the original and the approximated matrix on *x*-axis, which can be interpreted as the accuracy of the approximated values. In contrast, on *y*-axis we present the Euclidean norm of the difference between the corresponding rows in the centered original and centered approximated matrix, which can be interpreted as the average error of the reconstruction of the original pattern. We see that for each row (patient) the STMF’s value on *y*-axis is smaller than the NMF’s value, indicating that STMF better approximates the original patterns. The rows in the STMF’s approximation in Fig. 4 with predominantly low values have large approximation errors (*y*-axis) in Fig. 9 while having a comparable approximation of the original pattern as NMF’s approximation of the original pattern.

**Fig. 10:**
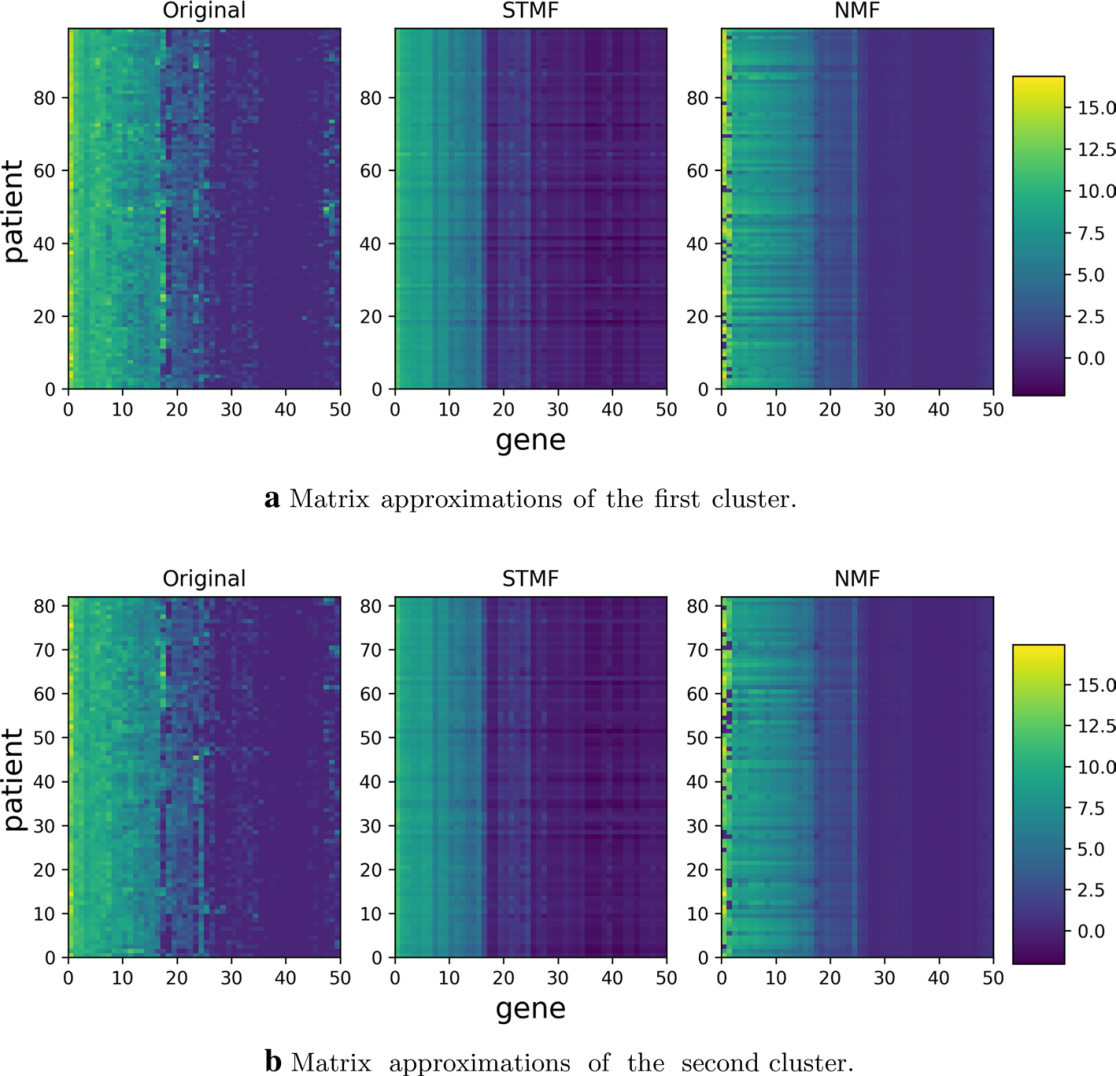
Comparison of STMF’s and NMF’s approximations of specific patients from two clusters generated by NMF, shown in Fig. 9. First cluster has a center positioned around (17, 141), while second cluster is positioned around (15, 109)

We see that NMF has two clusters of patients with large values on *y*-axis, denoted by red stars and red circles. These are the rows (patients) where the NMF’s predicted pattern differs significantly from the original pattern, more than the STMF’s predictions, but at the same time NMF is achieving smaller approximation error than STMF. In Fig. 10 we plot the patients corresponding to these two clusters and compare approximations with original data. It can be seen that NMF cannot model high (yellow) values in a few first columns, while low (blue) values are larger (light blue) compared to the original matrix, which has around half of the data plotted with dark blue. Comparison with Pearson and Spearman correlation is shown in Additional file 1: Figure S 25, where STMF achieves higher Pearson correlation, but lower Spearman correlation. Clusters of patients are also visible in both figures using these two correlations confirming results in Fig. 9. For all other datasets plots are available in Additional file 1, Section 2.

## Conclusion

Standard linear algebra is used in the majority of data mining and machine learning tasks. Utilizing different types of semirings has the potential to reveal previously undiscovered patterns. The motivation for using tropical semiring in matrix factorization methods is that resulting factors should give us the most dominant features that are specific and essential for each factor. In that way, factors are likely easier to interpret.

We propose a method called STMF, which can work with missing values. We implement STMF by extending TMF algorithm to be able to handle unknown values. Results show that NMF could not successfully recover the patterns on specific synthetic data, while the approximation with STMF achieves a higher correlation value. Results on TCGA data show that STMF outperforms NMF in the prediction task. Also, the results obtained by NMF tend toward the mean value, while the approximations obtained by STMF better express extreme values. Our proposed approach identifies strong patterns that aid the visual interpretation of results. In this way, we can discover sharp, high-variance components in the data. To the best of our knowledge, STMF is the first work using tropical semiring in sparse (biomedical) data.

A limitation of our STMF method is its apparent inability to embed and predict truly novel examples (i.e., new incomplete rows or columns in the data matrix). Developing an approach similar to the one we have shown for NMF [17] deserves further research to address this important task.

Another limitation of STMF method is the fact that can be used only on single data source. Integrative data fusion methods are based on co-factorization of multiple data matrices. Using standard linear algebra, DFMF is a variant of penalized matrix tri-factorization, which simultaneously factorizes data matrices to reveal hidden associations. It can model multiple relations between multiple object types, while relations between some object types can be completely missing. In our future work, we will investigate ways to modify the STMF method for data fusion of multiple data sources focusing on the fusion of methylation, miRNA, and gene expression data.

We believe that future research will show that semirings are useful in many scenarios and that they find the structure that is different and easier to interpret than with standard linear algebra.

## Supplementary Information

**Additional file 1: **Supplementary materials (Supplementary Figures S1–S72).

## Data Availability

This paper uses the real TCGA data available on http://acgt.cs.tau.ac.il/multi_omic_benchmark/download.html. PAM50 data can be found on the https://github.com/CSB-IG/pa3bc/tree/master/bioclassifier_R/. BIC subtypes are collected from https://www.cbioportal.org/. STMF code, PAM50 data and BIC subtypes are available on https://github.com/Ejmric/STMF.
