# Supplementary material for: Sparse data embedding and prediction by tropical matrix factorization
Source: BMC Bioinformatics. 2021 Feb 25;22:89. doi: 10.1186/s12859-021-04023-9 (PMC7908717; doi:10.1186/s12859-021-04023-9)
Supplement: Supplementary file 1 — Additional file 1: Supplementary materials (Supplementary Figures S1–S72). [file 12859_2021_4023_MOESM1_ESM.pdf]

Sparse data embedding and prediction by tropical matrix factorization: Supplementary Material

Amra Omanović, Hilal Kazan, Polona Oblak and Tomaž Curk

February 2, 2021

Contents

1 Synthetic data **2**

1.1 Effect of random sampling strategy of missing values 3

1.1.1 Missing at random 3

1.1.2 Missing not at random 5

1.2 Ordering techniques 7

2 Real data **8**

2.1 `BIC` 8

2.2 `AML` 12

2.3 `COLON` 14

2.4 `GBM` 16

2.5 `LIHC` 18

2.6 `LUSC` 20

2.7 `OV` 22

2.8 `SKCM` 24

2.9 `SARC` 26

1 Synthetic data

In Supplementary Figure S 1 we present original factor matrices of a smaller synthetic dataset. Approximation matrices of rank 4 are shown in Supplementary Figure S 2, while factor matrices are in Supplementary Figure S 3 and latent matrices in Supplementary Figure S 4.

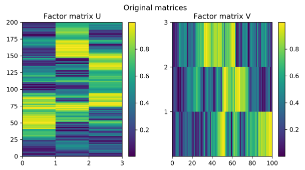

Supplementary Figure S 1: Original factor matrices.

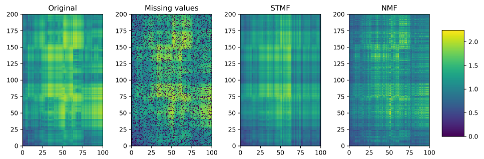

Supplementary Figure S 2: A comparison between STMF's and NMF's predictions of rank 4 approximations on $(200, 100)$ synthetic $(\max, +)$ matrix with 20% missing values.

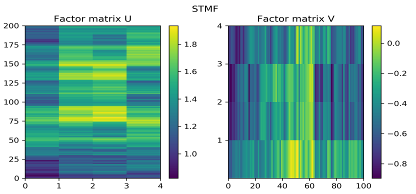

(a) Factor matrices $U_{\texttt{STMF}}, V_{\texttt{STMF}}$ from STMF

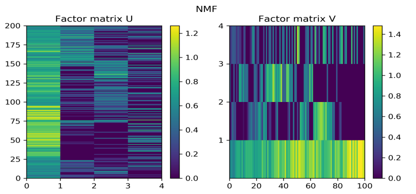

(b) Factor matrices $U_{\texttt{NMF}}, V_{\texttt{NMF}}$ from NMF

Supplementary Figure S 3: Factor matrices $U_{\texttt{STMF}}, V_{\texttt{STMF}}$ and $U_{\texttt{NMF}}, V_{\texttt{NMF}}$ from STMF and NMF, respectively.

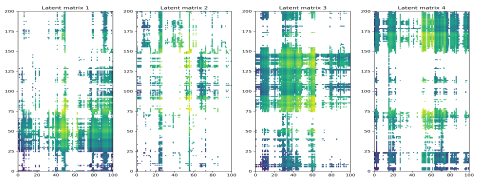

(a) Latent matrices $R^{(i)}_{\texttt{STMF}}$, $i \in \{1, 4\}$, where white represents the element which does not contribute to the approximation $R_{\texttt{STMF}}$.

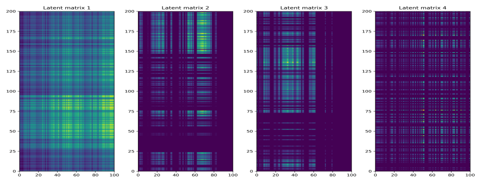

(b) Latent matrices $R^{(i)}_{\texttt{NMF}}$, $i \in \{1, 4\}$.

Supplementary Figure S 4: STMF's and NMF's latent matrices.

1.1 Effect of random sampling strategy of missing values

1.1.1 Missing at random

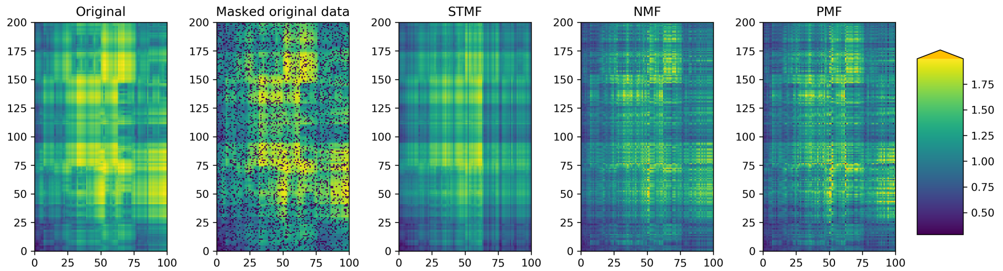

Supplementary Figure S 5: The experiment on synthetic dataset when the missing process is not correlated with the value itself, *i.e.* missing completely at random. Original data matrix has mean value of 1.336, `STMF`'s approximation 1.163, `NMF`'s and `PMF`'s approximations 1.093.

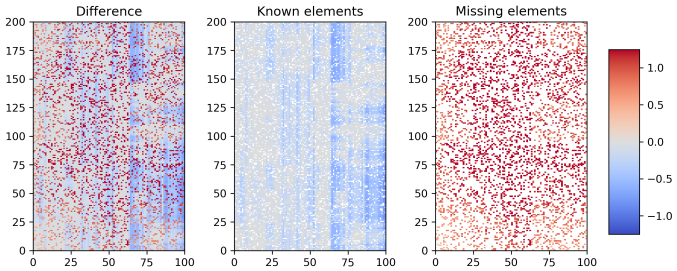

Supplementary Figure S 6: Difference between masked original data and `STMF`'s approximation.

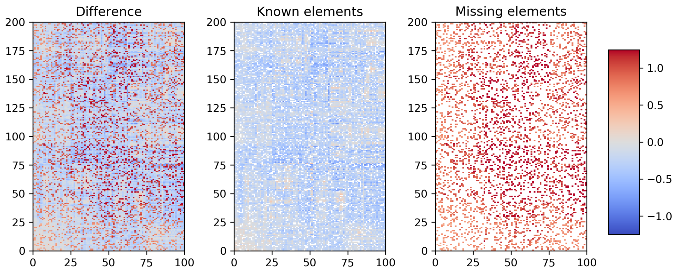

Supplementary Figure S 7: Difference between masked original data and `NMF`'s approximation.

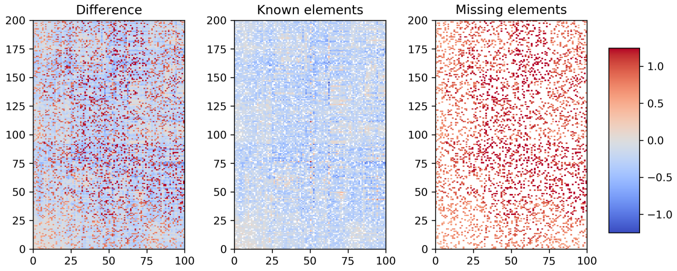

Supplementary Figure S 8: Difference between masked original data and PMF's approximation.

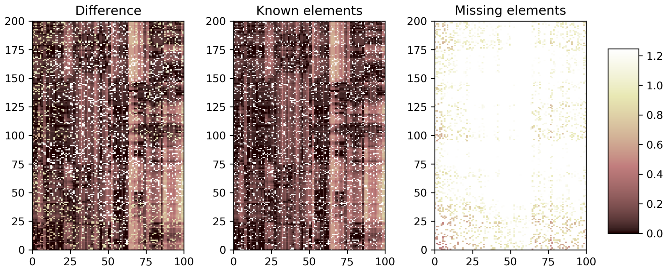

Supplementary Figure S 9: The absolute difference between masked original data and STMF's approximation.

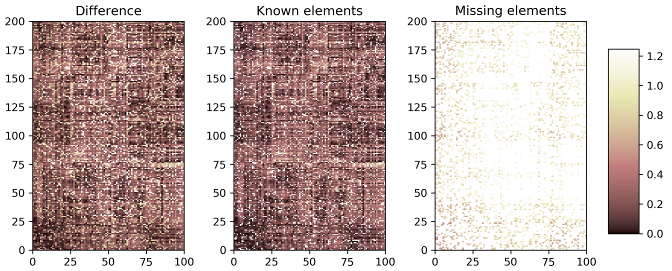

Supplementary Figure S 10: The absolute difference between masked original data and NMF's approximation.

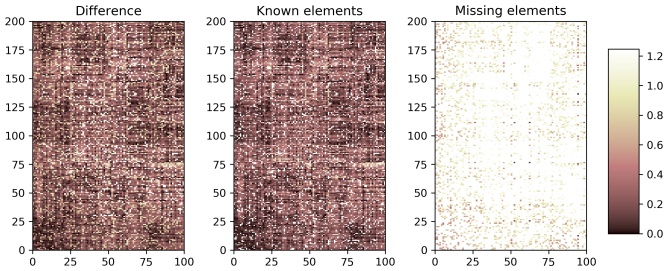

Supplementary Figure S 11: The absolute difference between masked original data and PMF's approximation.

1.1.2 Missing not at random

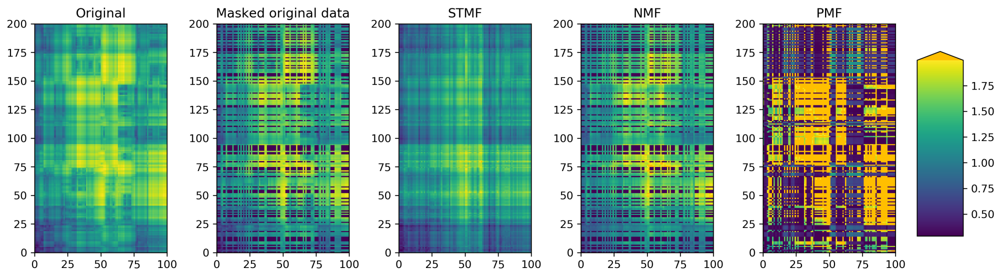

Supplementary Figure S 12: The experiment on synthetic dataset when the missing process is correlated with the value itself, *i.e.* missing not at random. Original data matrix has mean value of 1.336, `STMF`'s approximation 1.152, `NMF`'s approximation 1.074 and `PMF`'s approximation 1.070.

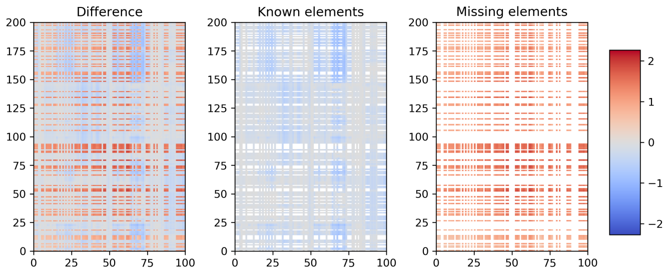

Supplementary Figure S 13: Difference between masked original data and `STMF`'s approximation.

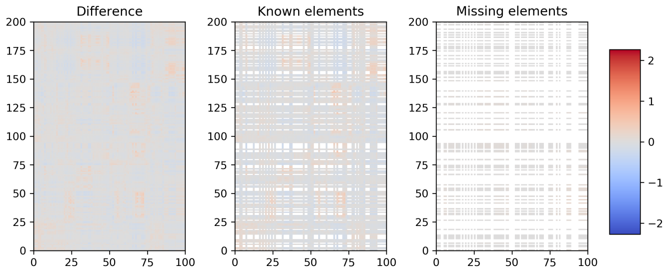

Supplementary Figure S 14: Difference between masked original data and `NMF`'s approximation.

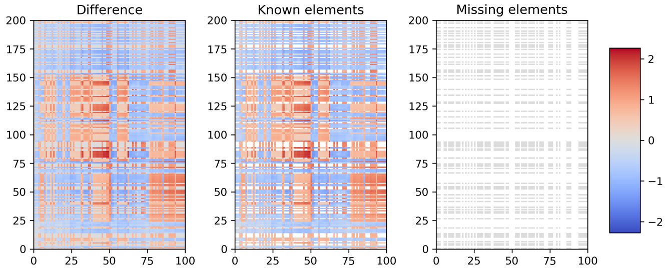

Supplementary Figure S 15: Difference between masked original data and `PMF`'s approximation.

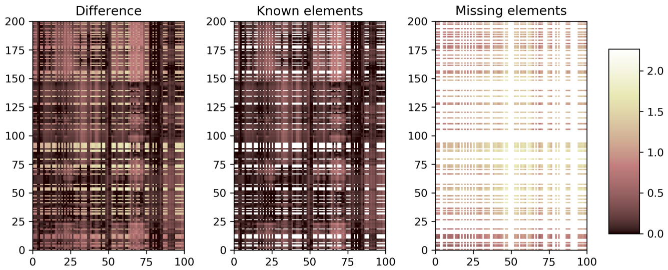

Supplementary Figure S 16: The absolute difference between masked original data and `STMF`'s approximation.

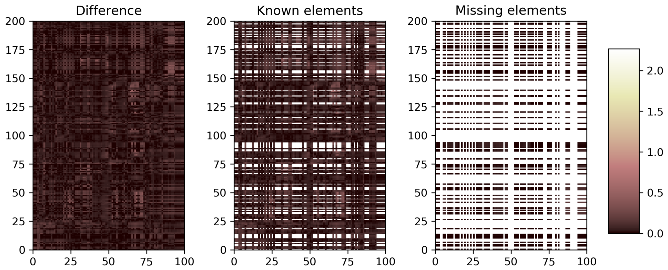

Supplementary Figure S 17: The absolute difference between masked original data and `NMF`'s approximation.

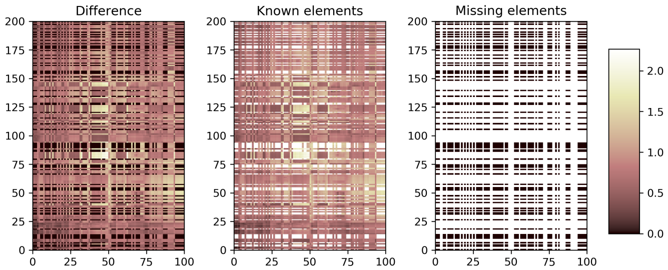

Supplementary Figure S 18: The absolute difference between masked original data and `PMF`'s approximation.

1.2 Ordering techniques

In Supplementary Figure S 19 we present five large synthetic datasets used for the ordering techniques experiment. Effect of different ordering strategies for these five datasets is shown in Supplementary Figure S 20.

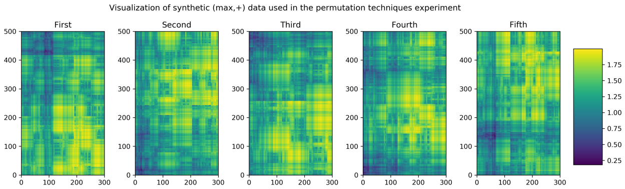

Supplementary Figure S 19: Synthetic data used in the ordering techniques experiment.

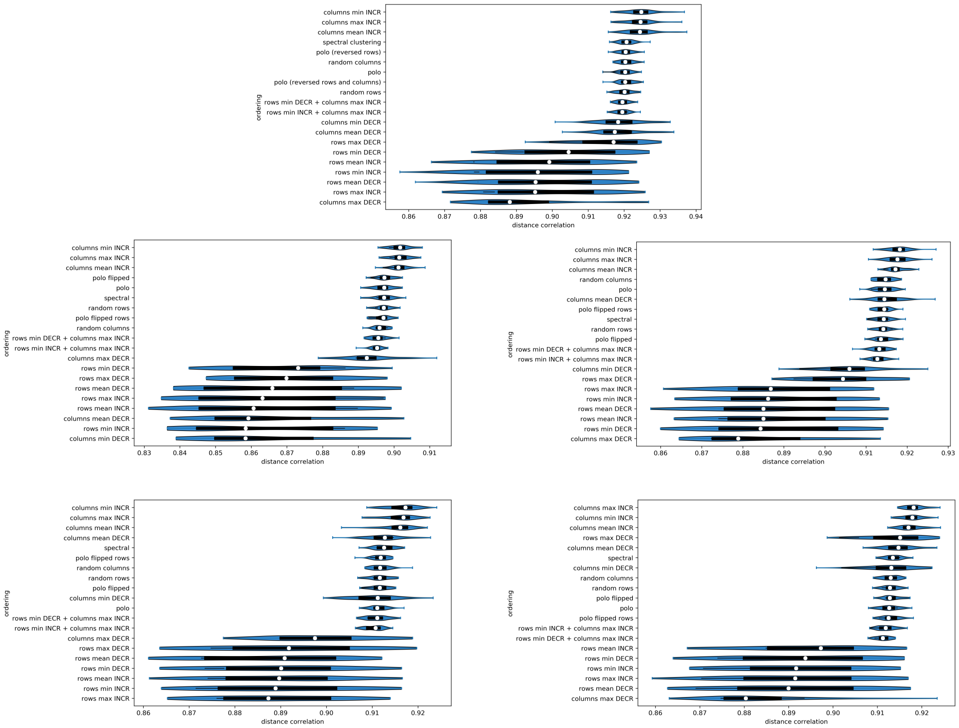

Supplementary Figure S 20: Effect of ordering strategy on achieved distance correlation by `STMF`, on five 500×300 synthetic $(\max, +)$ matrices from Figure 19.

2 Real data

In this section, we present results on real data using best approximation matrices of the corresponding rank. For the `STMF` method, we use Random Acol initialization described in the paper, and for the `NMF` method, we use a method `NNDSVD` [1] designed to enhance the initialization stage of `NMF`.

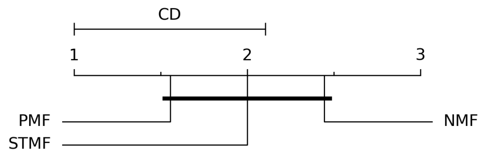

Supplementary Figure S 21: Critical difference (CD) [2] graph of average ranks of three methods (`STMF`, `NMF` and `PMF`) tested on nine datasets, for $\alpha = 0.05$. No statistically significant difference among the average ranks can be observed.

2.1 `BIC`

In Supplementary Figure S 22 we present the silhouette plot of `BIC` data which contains five clusters. Values can range from -1 to 1, where a value of 0 indicates that the sample (patient) is on or very close to the decision boundary between two neighboring clusters.

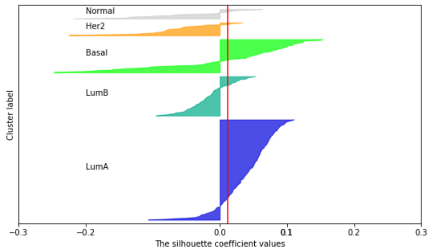

Supplementary Figure S 22: Silhouette plot of `BIC` gene expression data and five groups of `PAM50` (`BIC` subtypes) marker genes. The red line represents the average silhouette score (0.012).

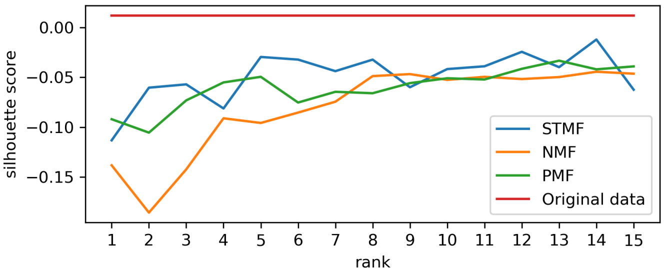

Supplementary Figure S 23: Comparison of silhouette scores between `STMF`, `NMF` and `PMF` obtained on `BIC` data and five groups of `PAM50` (`BIC` subtypes) marker genes.

In Supplementary Figure S 24, we plot distributions of original data and feature agglomeration data for all eight datasets.

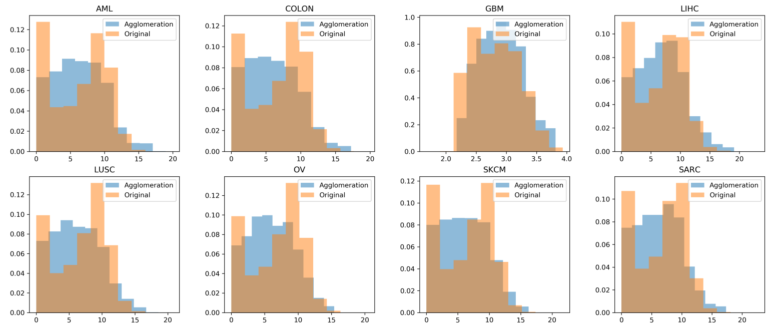

Supplementary Figure S 24: Distribution of original and feature agglomeration data.

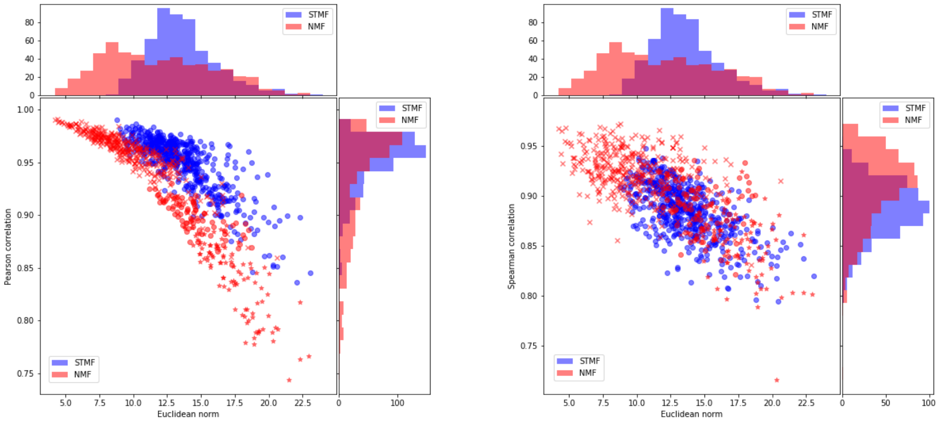

(a) Pearson correlation (b) Spearman correlation

Supplementary Figure S 25: Pearson and Spearman correlation on `BIC` data.

In Supplementary Figure S 25, we present Pearson and Spearman correlation results on `BIC` data.

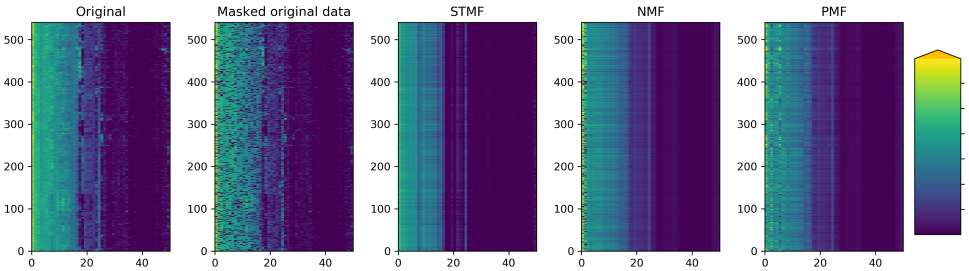

Supplementary Figure S 26: A comparison between `STMF`'s, `NMF`'s and `PMF`'s predictions of rank 3 approximations on `BIC` data with 20% missing values.

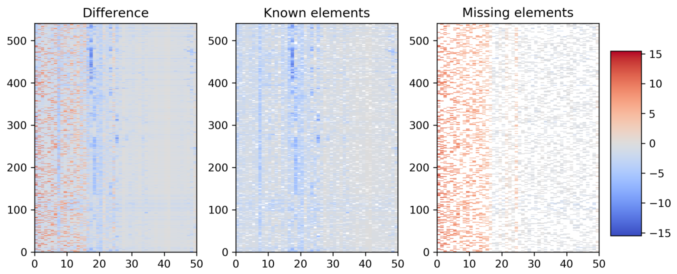

Supplementary Figure S 27: Difference between masked original data and STMF's approximation.

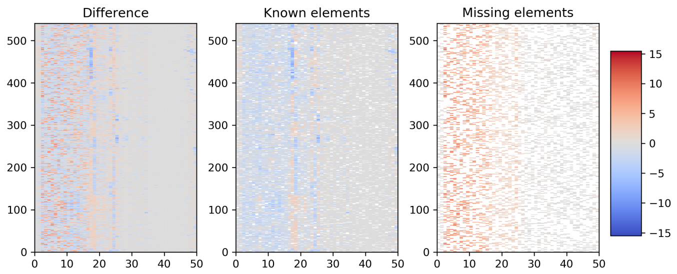

Supplementary Figure S 28: Difference between masked original data and NMF's approximation.

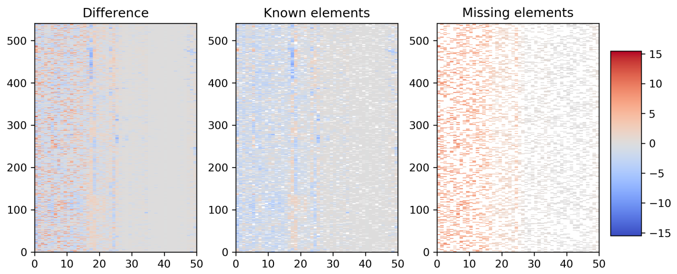

Supplementary Figure S 29: Difference between masked original data and PMF's approximation.

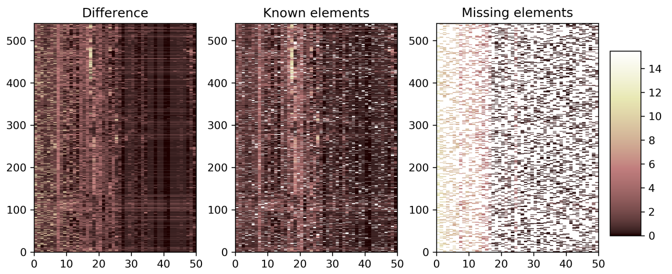

Supplementary Figure S 30: The absolute difference between masked original data and STMF's approximation.

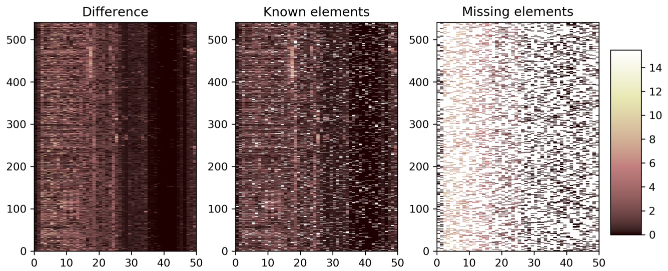

Supplementary Figure S 31: The absolute difference between masked original data and `NMF`'s approximation.

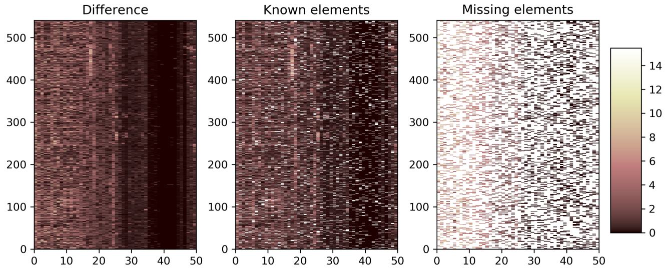

Supplementary Figure S 32: The absolute difference between masked original data and `PMF`'s approximation.

2.2 AML

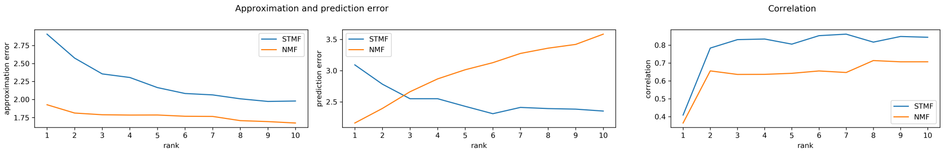

Supplementary Figure S 33: Difference between approximation and prediction RMSE and distance correlation of STMF and NMF on AML data.

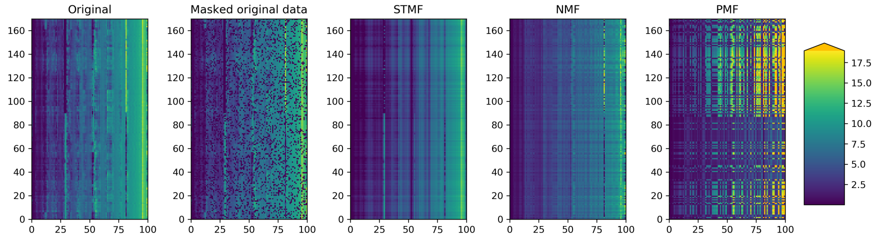

Supplementary Figure S 34: A comparison between STMF's, NMF's and PMF's predictions of rank 3 approximations on AML data with 20% missing values.

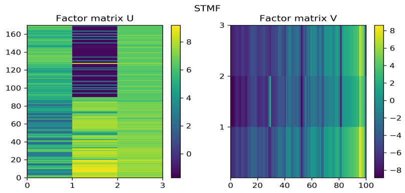

(a) Factor matrices $U_{\texttt{STMF}}, V_{\texttt{STMF}}$ from STMF.

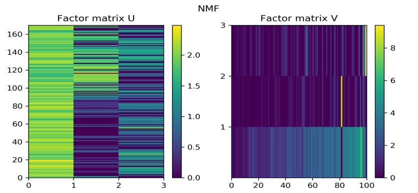

(b) Factor matrices $U_{\texttt{NMF}}, V_{\texttt{NMF}}$ from NMF.

Supplementary Figure S 35: Factor matrices $U_{\texttt{STMF}}, V_{\texttt{STMF}}$ and $U_{\texttt{NMF}}, V_{\texttt{NMF}}$ from STMF and NMF on AML data, respectively.

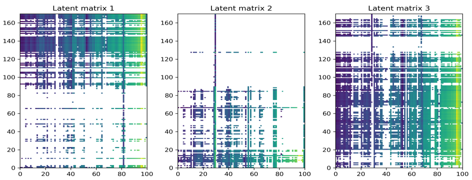

(a) Latent matrices $R^{(i)}_{\texttt{STMF}}$, $i \in \{1, 3\}$, where white represents the element which does not contribute to the approximation $R_{\texttt{STMF}}$.

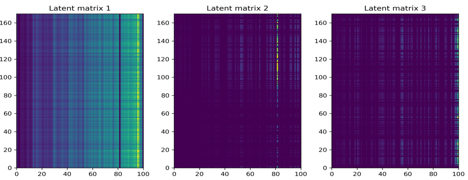

(b) Latent matrices $R^{(i)}_{\texttt{NMF}}$, $i \in \{1, 3\}$.

Supplementary Figure S 36: STMF's and NMF's latent matrices on AML data.

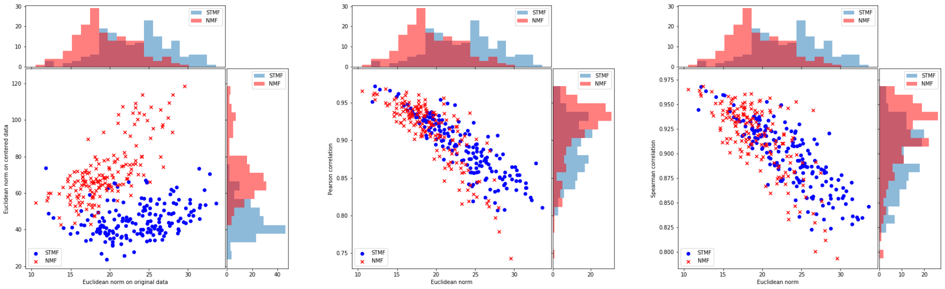

(a) Euclidean norm on centered data

(b) Pearson correlation

(c) Spearman correlation

Supplementary Figure S 37: Euclidean norm on centered data, Pearson and Spearman correlation on `AML` data.

2.3 COLON

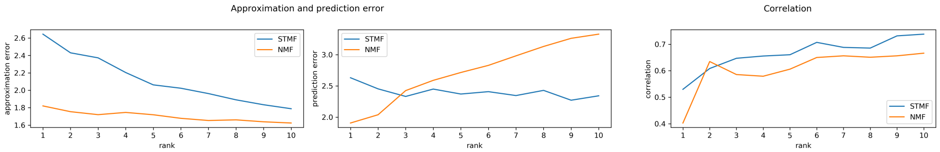

Supplementary Figure S 38: Difference between approximation and prediction RMSE and distance correlation of STMF and NMF on COLON data.

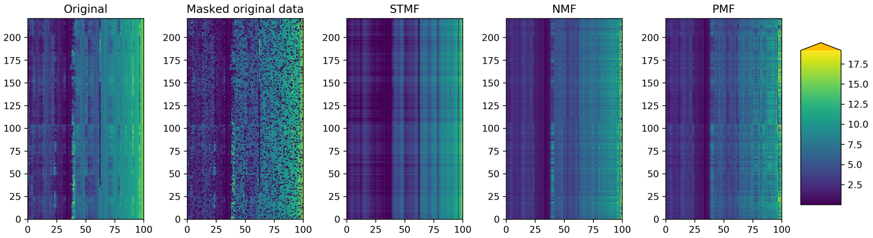

Supplementary Figure S 39: A comparison between STMF's, NMF's and PMF's predictions of rank 3 approximations on COLON data with 20% missing values.

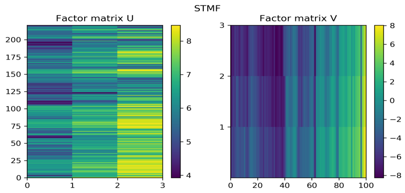

(a) Factor matrices $U_{\texttt{STMF}}, V_{\texttt{STMF}}$ from STMF.

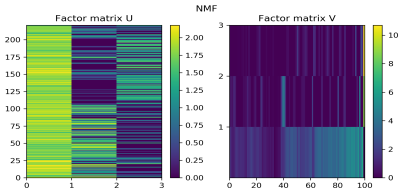

(b) Factor matrices $U_{\texttt{NMF}}, V_{\texttt{NMF}}$ from NMF.

Supplementary Figure S 40: Factor matrices $U_{\texttt{STMF}}, V_{\texttt{STMF}}$ and $U_{\texttt{NMF}}, V_{\texttt{NMF}}$ from STMF and NMF on COLON data, respectively.

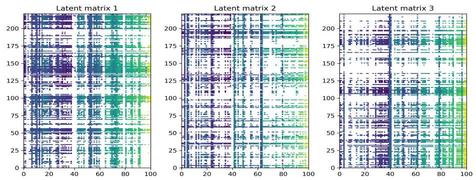

(a) Latent matrices $R^{(i)}_{\texttt{STMF}}$, $i \in \{1, 3\}$, where white represents the element which does not contribute to the approximation $R_{\texttt{STMF}}$.

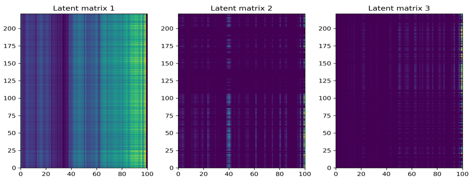

(b) Latent matrices $R^{(i)}_{\texttt{NMF}}$, $i \in \{1, 3\}$.

Supplementary Figure S 41: STMF's and NMF's latent matrices on COLON data.

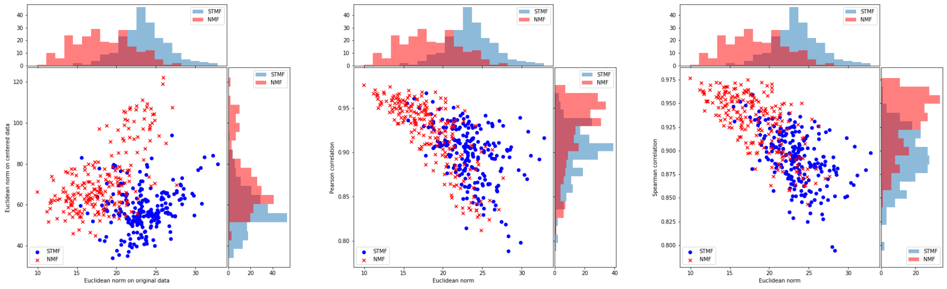

(a) Euclidean norm on centered data (b) Pearson correlation (c) Spearman correlation

Supplementary Figure S 42: Euclidean norm on centered data, Pearson and Spearman correlation on `COLON` data.

2.4 GBM

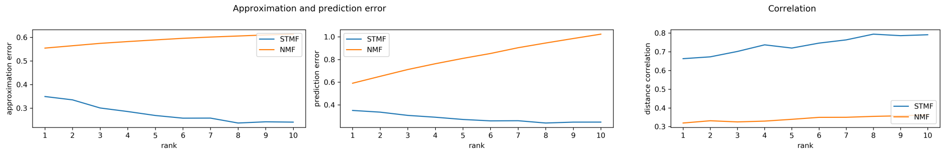

Supplementary Figure S 43: Difference between approximation and prediction RMSE and distance correlation of `STMF` and `NMF` on `GBM` data.

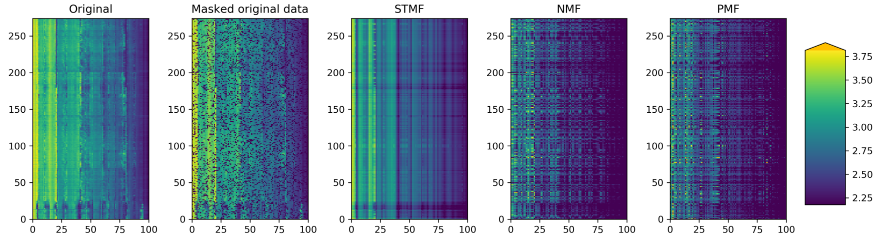

Supplementary Figure S 44: A comparison between `STMF`'s, `NMF`'s and `PMF`'s predictions of rank 3 approximations on `GBM` data with 20% missing values.

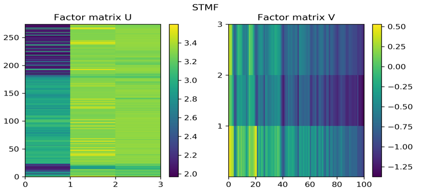

(a) Factor matrices $U_{\texttt{STMF}}, V_{\texttt{STMF}}$ from `STMF`.

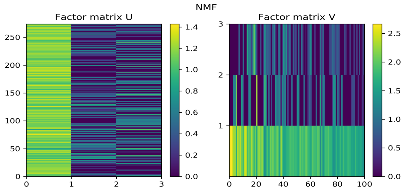

(b) Factor matrices $U_{\texttt{NMF}}, V_{\texttt{NMF}}$ from `NMF`.

Supplementary Figure S 45: Factor matrices $U_{\texttt{STMF}}, V_{\texttt{STMF}}$ and $U_{\texttt{NMF}}, V_{\texttt{NMF}}$ from `STMF` and `NMF` on `GBM` data, respectively.

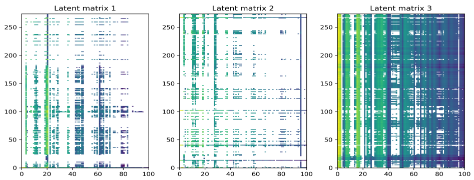

(a) Latent matrices $R^{(i)}_{\texttt{STMF}}$, $i \in \{1, 3\}$, where white represents the element which does not contribute to the approximation $R_{\texttt{STMF}}$.

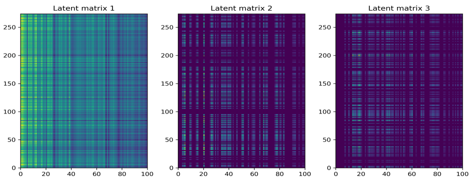

(b) Latent matrices $R^{(i)}_{\texttt{NMF}}$, $i \in \{1, 3\}$.

Supplementary Figure S 46: `STMF`'s and `NMF`'s latent matrices on `GBM` data.

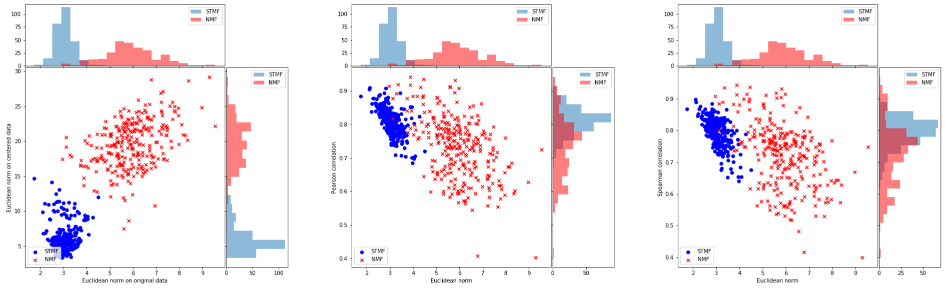

(a) Euclidean norm on centered data

(b) Pearson correlation

(c) Spearman correlation

Supplementary Figure S 47: Euclidean norm on centered data, Pearson and Spearman correlation on `GBM` data.

2.5 LIHC

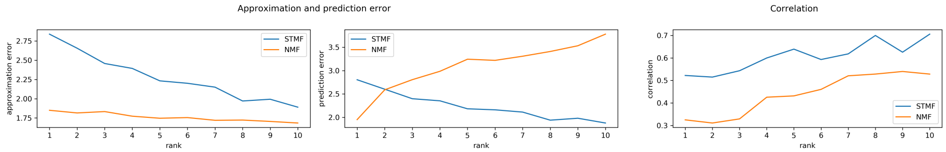

Supplementary Figure S 48: Difference between approximation and prediction RMSE and distance correlation of STMF and NMF on LIHC data.

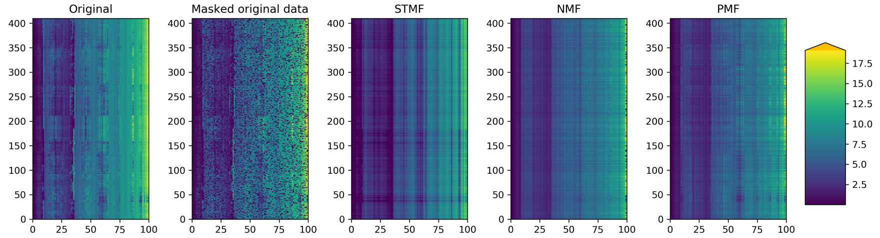

Supplementary Figure S 49: A comparison between STMF's, NMF's and PMF's predictions of rank 2 approximations on LIHC data with 20% missing values.

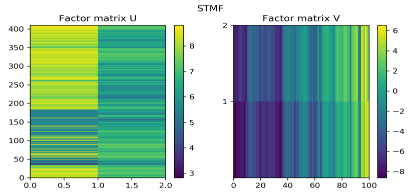

(a) Factor matrices $U_{\texttt{STMF}}, V_{\texttt{STMF}}$ from STMF.

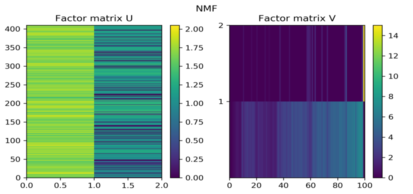

(b) Factor matrices $U_{\texttt{NMF}}, V_{\texttt{NMF}}$ from NMF.

Supplementary Figure S 50: Factor matrices $U_{\texttt{STMF}}, V_{\texttt{STMF}}$ and $U_{\texttt{NMF}}, V_{\texttt{NMF}}$ from STMF and NMF on LIHC data, respectively.

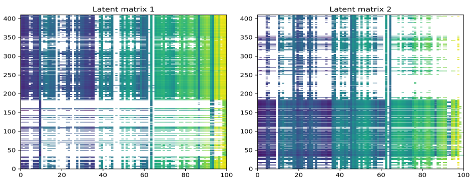

(a) Latent matrices $R^{(i)}_{\texttt{STMF}}$, $i \in \{1, 2\}$, where white represents the element which does not contribute to the approximation $R_{\texttt{STMF}}$.

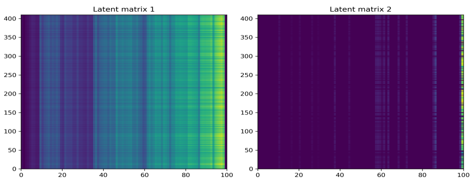

(b) Latent matrices $R^{(i)}_{\texttt{NMF}}$, $i \in \{1, 2\}$.

Supplementary Figure S 51: STMF's and NMF's latent matrices on LIHC data.

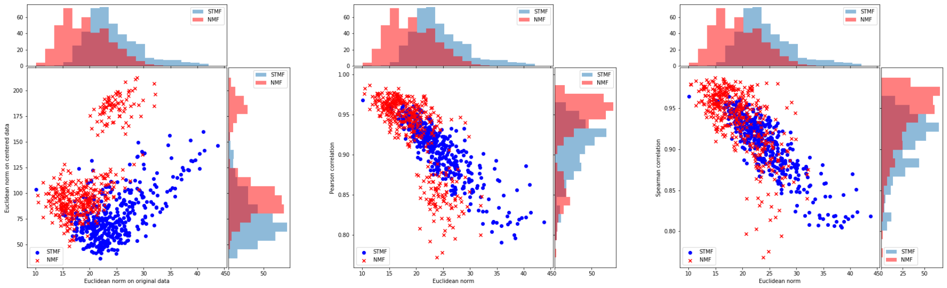

(a) Euclidean norm on centered data

(b) Pearson correlation

(c) Spearman correlation

Supplementary Figure S 52: Euclidean norm on centered data, Pearson and Spearman correlation on `LIHC` data.

2.6 LUSC

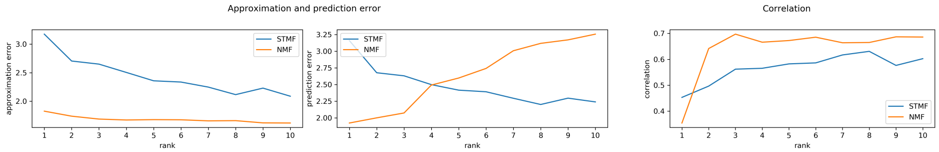

Supplementary Figure S 53: Difference between approximation and prediction RMSE and distance correlation of STMF and NMF on LUSC data.

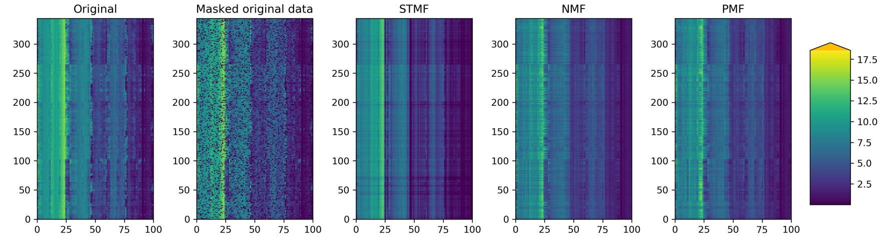

Supplementary Figure S 54: A comparison between STMF's, NMF's and PMF's predictions of rank 3 approximations on LUSC data with 20% missing values.

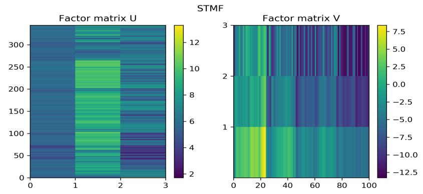

(a) Factor matrices $U_{\mathtt{STMF}}, V_{\mathtt{STMF}}$ from STMF.

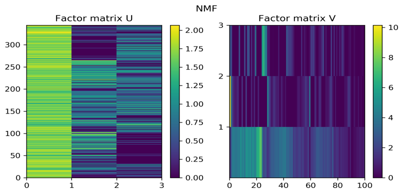

(b) Factor matrices $U_{\mathtt{NMF}}, V_{\mathtt{NMF}}$ from NMF.

Supplementary Figure S 55: Factor matrices $U_{\mathtt{STMF}}, V_{\mathtt{STMF}}$ and $U_{\mathtt{NMF}}, V_{\mathtt{NMF}}$ from STMF and NMF on LUSC data, respectively.

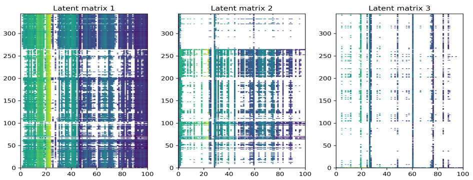

(a) Latent matrices $R^{(i)}_{\mathtt{STMF}}$, $i \in \{1, 3\}$, where white represents the element which does not contribute to the approximation $R_{\mathtt{STMF}}$.

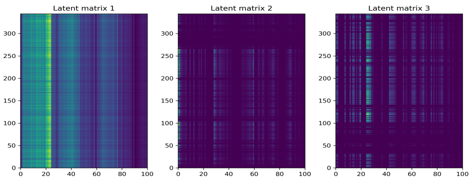

(b) Latent matrices $R^{(i)}_{\mathtt{NMF}}$, $i \in \{1, 3\}$.

Supplementary Figure S 56: STMF's and NMF's latent matrices on LUSC data.

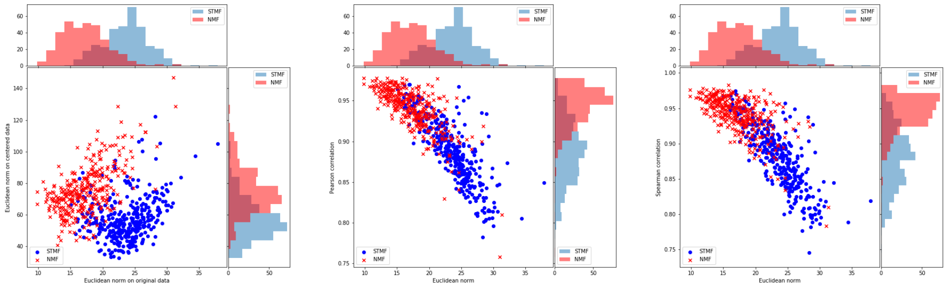

(a) Euclidean norm on centered data (b) Pearson correlation (c) Spearman correlation

Supplementary Figure S 57: Euclidean norm on centered data, Pearson and Spearman correlation on `LUSC` data.

2.7 OV

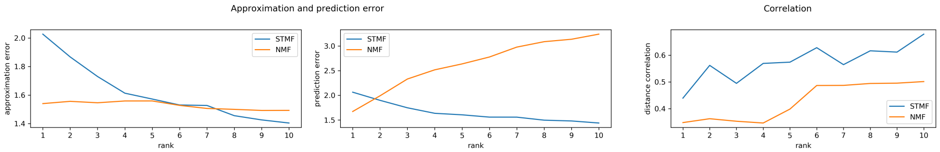

Supplementary Figure S 58: Difference between approximation and prediction RMSE and distance correlation of STMF and NMF on OV data.

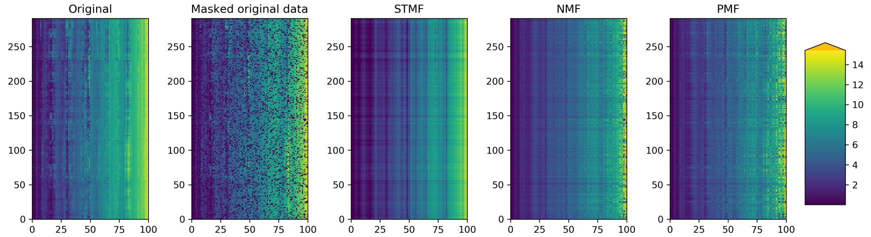

Supplementary Figure S 59: A comparison between STMF's, NMF's and PMF's predictions of rank 4 approximations on OV data with 20% missing values.

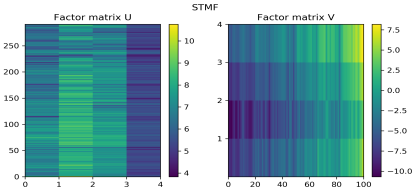

(a) Factor matrices $U_{\texttt{STMF}}, V_{\texttt{STMF}}$ from STMF.

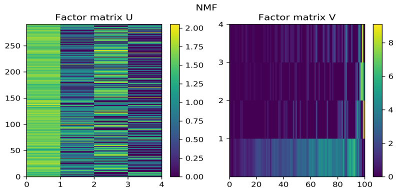

(b) Factor matrices $U_{\texttt{NMF}}, V_{\texttt{NMF}}$ from NMF.

Supplementary Figure S 60: Factor matrices $U_{\texttt{STMF}}, V_{\texttt{STMF}}$ and $U_{\texttt{NMF}}, V_{\texttt{NMF}}$ from STMF and NMF on OV data, respectively.

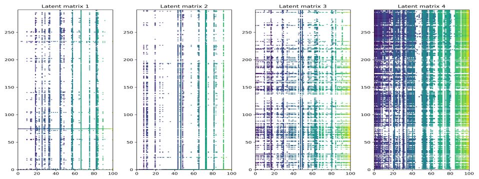

(a) Latent matrices $R^{(i)}_{\texttt{STMF}}$, $i \in \{1, 4\}$, where white represents the element which does not contribute to the approximation $R_{\texttt{STMF}}$.

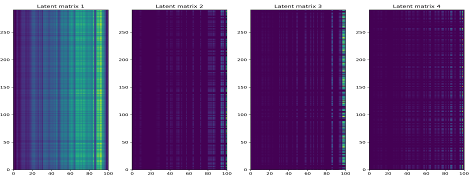

(b) Latent matrices $R^{(i)}_{\texttt{NMF}}$, $i \in \{1, 4\}$.

Supplementary Figure S 61: STMF's and NMF's latent matrices on OV data.

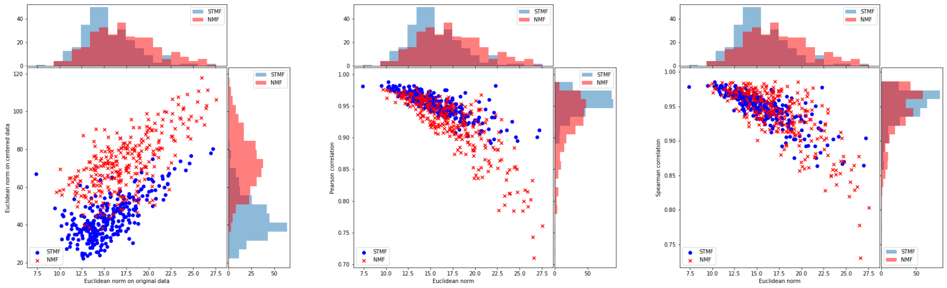

(a) Euclidean norm on centered data

(b) Pearson correlation

(c) Spearman correlation

Supplementary Figure S 62: Euclidean norm on centered data, Pearson and Spearman correlation on `OV` data.

2.8 SKCM

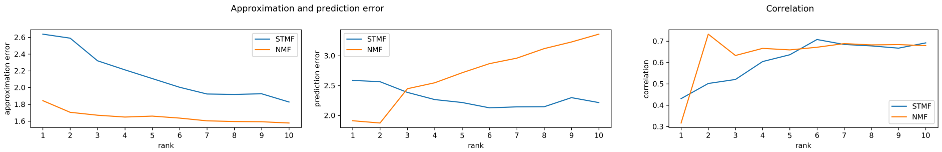

Supplementary Figure S 63: Difference between approximation and prediction RMSE and distance correlation of STMF and NMF on SKCM data.

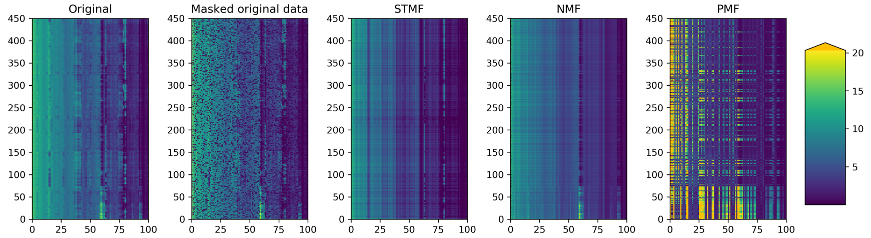

Supplementary Figure S 64: A comparison between STMF's, NMF's and PMF's predictions of rank 3 approximations on SKCM data with 20% missing values.

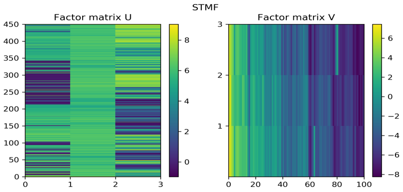

(a) Factor matrices $U_{\texttt{STMF}}, V_{\texttt{STMF}}$ from STMF.

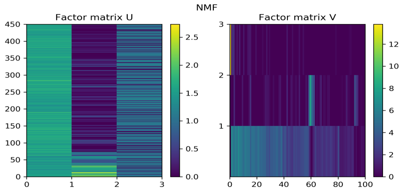

(b) Factor matrices $U_{\texttt{NMF}}, V_{\texttt{NMF}}$ from NMF.

Supplementary Figure S 65: Factor matrices $U_{\texttt{STMF}}, V_{\texttt{STMF}}$ and $U_{\texttt{NMF}}, V_{\texttt{NMF}}$ from STMF and NMF on SKCM data, respectively.

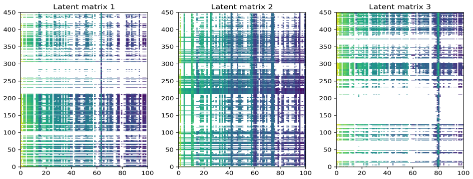

(a) Latent matrices $R^{(i)}_{\texttt{STMF}}$, $i \in \{1, 3\}$, where white represents the element which does not contribute to the approximation $R_{\texttt{STMF}}$.

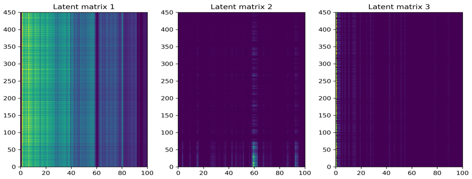

(b) Latent matrices $R^{(i)}_{\texttt{NMF}}$, $i \in \{1, 3\}$.

Supplementary Figure S 66: STMF's and NMF's latent matrices on SKCM data.

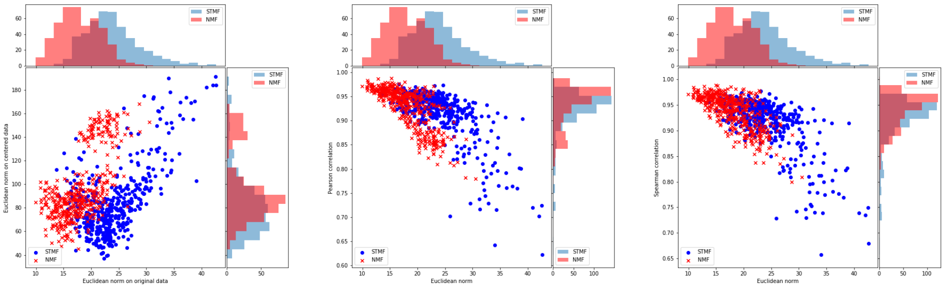

(a) Euclidean norm on centered data

(b) Pearson correlation

(c) Spearman correlation

Supplementary Figure S 67: Euclidean norm on centered data, Pearson and Spearman correlation on `SKCM` data.

2.9 SARC

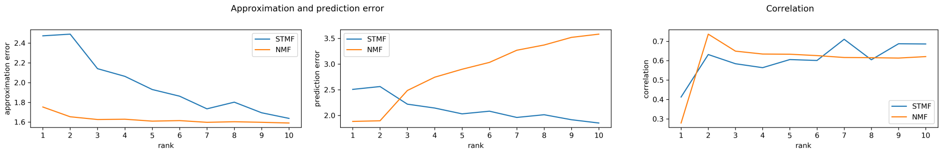

Supplementary Figure S 68: Difference between approximation and prediction RMSE and distance correlation of STMF and NMF on SARC data.

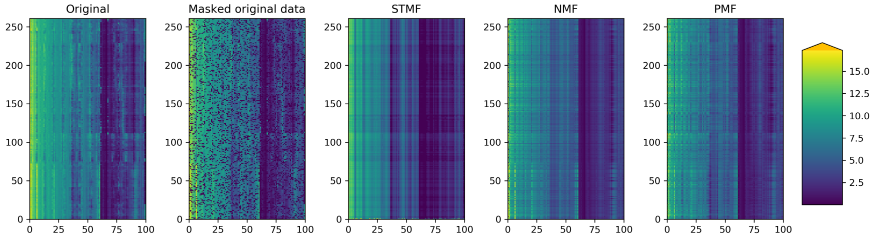

Supplementary Figure S 69: A comparison between STMF's, NMF's and PMF's predictions of rank 3 approximations on SARC data with 20% missing values.

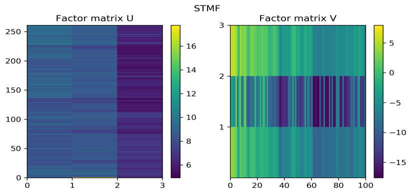

(a) Factor matrices $U_{\texttt{STMF}}, V_{\texttt{STMF}}$ from STMF.

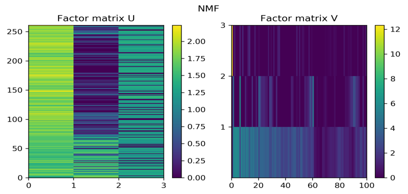

(b) Factor matrices $U_{\texttt{NMF}}, V_{\texttt{NMF}}$ from NMF.

Supplementary Figure S 70: Factor matrices $U_{\texttt{STMF}}, V_{\texttt{STMF}}$ and $U_{\texttt{NMF}}, V_{\texttt{NMF}}$ from STMF and NMF on SARC data, respectively.

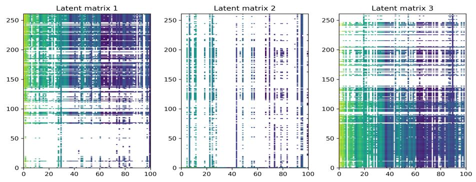

(a) Latent matrices $R^{(i)}_{\texttt{STMF}}$, $i \in \{1, 3\}$, where white represents the element which does not contribute to the approximation $R_{\texttt{STMF}}$.

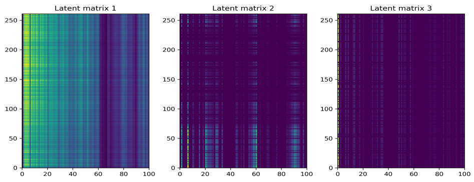

(b) Latent matrices $R^{(i)}_{\texttt{NMF}}$, $i \in \{1, 3\}$.

Supplementary Figure S 71: STMF's and NMF's latent matrices on SARC data.

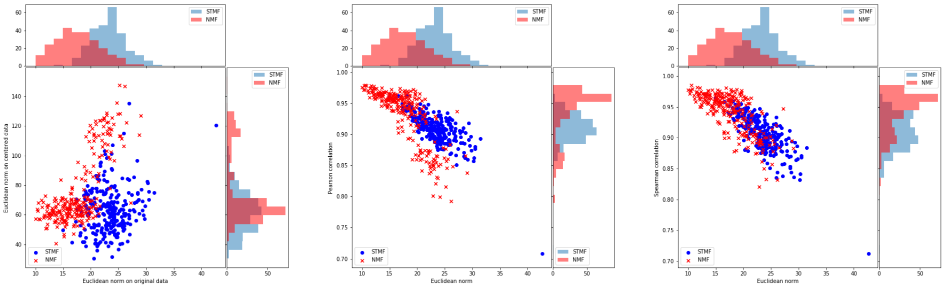

(a) Euclidean norm on centered data

(b) Pearson correlation

(c) Spearman correlation

Supplementary Figure S 72: Euclidean norm on centered data, Pearson and Spearman correlation on `SARC` data.

Author details

References

1. Boutsidis, C., Gallopoulos, E.: Svd based initialization: A head start for nonnegative matrix factorization. Pattern recognition **41**(4), 1350–1362 (2008)
2. Demšar, J.: Statistical comparisons of classifiers over multiple data sets. Journal of Machine learning research **7**(Jan), 1–30 (2006)
